# Dynamic single-cell landscape reveals a T-cell-suppressed microenvironment and a conserved neutrophil program in the lung metastatic niche

**DOI:** 10.1016/j.gendis.2026.102214

**Published:** 2026-04-27

**Authors:** Chen Ai, Huijuan Wu, Huihui Yang, Jing Wang, Chang Liu, Melissa S.F. Ng, Ziheng Zhou, Fenghui Zhuang, Qinhang Gao, Lingqian Wang, Changming Shi, Linnan Zhu, Zhaode Bu, Chang Chen, Lai Guan Ng, Zemin Zhang

**Affiliations:** a Biomedical Pioneering Innovative Center (BIOPIC), Academy for Advanced Interdisciplinary Studies, School of Life Sciences, State Key Laboratory of Metabolic Dysregulation & Prevention and Treatment of Esophageal Cancer, Peking University, Beijing 100871, China; b Beijing Advanced Center of Cellular Homeostasis and Aging-Related Diseases, Institute of Advanced Clinical Medicine, Peking University, Beijing 100191, China; c Institute for Translational Medicine on Cell Fate and Disease, Shanghai Ninth People's Hospital, Key Laboratory of Cell Differentiation and Apoptosis of the National Ministry of Education, Department of Pathophysiology, Shanghai Jiao Tong University School of Medicine, Shanghai 200025, China; d Department of Thoracic Surgery, Shanghai Pulmonary Hospital, School of Medicine, Tongji University, Shanghai 200433, China; e Shanghai Engineering Research Center of Lung Transplantation, Shanghai 200433, China; f Department of General Surgery, First Medical Center of Chinese PLA General Hospital, Beijing 100039, China; g Singapore Immunology Network (SIgN), Agency for Science, Technology and Research (A∗STAR), 138648, Singapore; h Changping Laboratory, Beijing 102206, China; i Department of Gastrointestinal Cancer Center, Peking University Cancer Hospital & Institute, Beijing 100142, China; j School of Medicine, Westlake University, Hangzhou, Zhejiang 310030, China; k Shanghai Immune Therapy Institute, Shanghai Jiao Tong University School of Medicine affiliated Renji Hospital, Shanghai 200127, China; l Chongqing Medical University, Chongqing 400016, China

**Keywords:** Circulating biomarkers, Lung metastasis, Metastatic niche, Neutrophil plasticity, Single-cell RNA-seq, T cell suppression

## Abstract

While the immune landscape of primary tumors is increasingly understood, the dynamic immune remodeling within the evolving lung metastatic microenvironment remains poorly characterized. Here, by systematically comparing the microenvironments of primary and metastatic tumors using single-cell transcriptomics across diverse cancer models, we reveal that lung metastases consistently establish a profoundly T-cell-suppressed microenvironment. Concurrently, we identified a strictly metastasis-specific neutrophil gene program. Crucially, this program is highly concurrent with the metastatic microenvironment and is fundamentally distinct from neutrophil phenotypes observed in primary tumors or healthy lungs. Among the core genes defining this program, we identified the cell surface marker STEAP4 as a highly specific indicator of these metastasis-associated neutrophils. In both murine models and human patients, these STEAP4^+^ neutrophils were not only enriched within the local lung metastatic niche but also detectable in the circulation. Furthermore, the abundance of circulating STEAP4^+^ neutrophils discriminates breast cancer patients with lung metastasis from those with localized primary tumors or healthy donors. Our findings delineate the divergent immune remodeling trajectories of lung metastasis and highlight their association with STEAP4^+^ neutrophils, establishing a useful biological framework for future functional and mechanistic investigations.

## Introduction

Metastasis remains the leading cause of cancer-related mortality, with the lungs acting as one of the most frequent target organs for tumor dissemination.^1^, ^2^, ^3^, ^4^ During this process, the lung tissue undergoes profound changes to establish a metastatic tumor microenvironment (TME) whose cellular and molecular characteristics differ fundamentally from those of primary tumors.^5^, ^6^, ^7^ While the immune landscape of primary tumors has been extensively mapped in recent years, the dynamic immune remodeling that occurs within the evolving lung metastatic niche remains poorly characterized. When viewing the lung as a shared host organ, it remains unclear whether metastases of diverse origins harbor conserved immune features and how their overall immune landscapes diverge from primary lung tumors. Currently, tracking metastatic progression largely relies on imaging modalities^8^ or the detection of circulating tumor cells, which often suffer from low sensitivity due to their rarity in the bloodstream.^8^^,^^9^ Investigating the dynamic immune remodeling within the local metastatic niche could provide critical biological insights, which may potentially unveil conceptually distinct avenues for metastatic disease monitoring.

For a long time, neutrophils in both primary and metastatic tumors were viewed as a short-lived, homogenous population.^10^^,^^11^ Recently, however, increasing studies have demonstrated that they can differentiate into specific phenotypes to adapt to local tissue microenvironments, including the primary TME.^12^, ^13^, ^14^, ^15^ However, whether neutrophils exhibit the same phenotypes in the metastatic niche as they do in the primary tumor remains poorly characterized. A key challenge in answering this question is that neutrophils in the metastatic niche may be influenced by organ-intrinsic properties, signals directly from the primary tumor, or both.

To address these gaps, we utilized single-cell transcriptomics to dynamically profile the immune landscapes of normal lungs, primary lung tumors, and lung metastases across multiple pre-clinical cancer models. We have built a user-friendly interactive website (http://lungmet.cancer-pku.cn/) to help researchers seamlessly explore and utilize our data. Additionally, we discovered that lung metastases from diverse primary origins exhibited a propensity to establish a T-cell-suppressed microenvironment. Concurrently, by systematically comparing neutrophil heterogeneity across steady-state, primary tumor, and lung metastatic conditions, we determined a highly conserved, metastasis-specific gene program. Among the core genes defining this program, the cell surface protein, STEAP (six transmembrane epithelial antigen of prostate) family member 4 (STEAP4) emerged as a highly specific indicator of these metastasis-associated neutrophils. Remarkably, in both murine models across diverse cancer types and human patients, these STEAP4^+^ neutrophils were not only enriched in the lung metastatic niche but also robustly detectable in the peripheral blood. Consistent with these local alterations, validation in murine cohorts demonstrated that the emergence of STEAP4^+^ neutrophils strictly co-occurred with lung metastasis. Furthermore, a preliminary evaluation in a small clinical cohort revealed that circulating STEAP4^+^ neutrophils were specifically enriched in breast cancer patients with lung metastasis, distinguishing them from healthy donors and individuals with localized primary tumors. Together, our findings delineate the divergent immune remodeling trajectories of lung metastasis and highlight neutrophil plasticity—specifically the emergence of STEAP4^+^ neutrophils—as a potential circulating indicator associated with this distinct immunosuppressive microenvironment in the context of cancer progression.

## Materials and methods

### Human subjects

All procedures involving study participants were approved by the Human Research Ethics Committee of the Shanghai Pulmonary Hospital (Approval No. K24-581). Written informed consent was obtained from all participants. Clinical information of all subjects in this study was listed in Table S1 (tissue sections) and Table S5 (blood samples for fluorescence-activated cell sorting (FACS)). All recruited patients were strictly screened to exclude concurrent acute infectious diseases (*e.g.*, pneumonia), chronic active inflammatory conditions, or severe trauma.

### Computed tomography (CT)

All the donors and patients underwent thin-section CT. All CT examinations were performed with a 64-section scanner (Somatom Definition AS+, Biograph64), Philips (Brilliance 40, iCT256, Ingenuity Flex, MX 16-slice) with contrast material, and United Imaging (uCT 510, uCT 760, uCT S-160). Chest CT lesions in each patient were identified by pathologists.

### Histology of human lung tissues

Human adult lung tissue samples were obtained from lung adenocarcinoma (LUAD) and breast cancer patients at the time of surgery. Additionally, normal lung tissue from adult donor lungs unsuitable for transplant and lung adenocarcinoma tissues from lobectomies were included. Fresh tissues were fixed with 4% paraformaldehyde at 4 °C overnight, and paraffin sections (4 μm) were prepared for immunofluorescent analysis and hematoxyli–eosin staining.

For the hematoxyli–eosin staining experiment followed our previous study.^16^ Briefly, slides were deparaffinized and rehydrated. The nuclei were stained with hematoxylin (Abcam, ab150678) for 2 min, and the cytoplasm was stained with eosin (Sigma, HT110280) for 3 min. Slices were then sealed with neutral resin after dehydration and clearing.

For immunofluorescent analysis, slides were deparaffinized and rehydrated, then washed three times (10 min each) with phosphate-buffered saline (PBS). A ring was drawn around the tissue using a hydrophobic pen (Vector, cat. no. H-4000). Slides were washed three times (10 min each) with PBS. Tissue was permeabilized with PBS-0.1% TritonX-100 (Sigma–Aldrich, cat. no. T9284-100 mL) for 10 min. Next, slides were washed three times (10 min each) with PBS. The slides were incubated with a blocking buffer consisting of 3% bovine serum albumin (Biofroxx, 4240GR100), 0.1% Tween-20, and 10% normal donkey serum (Servicebio, cat.no. G1217-5 ML) filtered through a 0.22-um filter, for 1 h in a humidified chamber. Primary antibodies (rabbit anti-CD8a, Abcam, ab237709; mouse anti-PD-1, Servicebio, GB12338; mouse anti-CD15, Abcam, ab241552; mouse anti-CD66b, GeneTex, GTX19779; rabbit anti-CD16, Abclonal, A23541; rabbit anti-STEAP4, Proteintech,11944-1-AP) were diluted in antibody dilution buffer (Ai Fang Biological, AFIHC014) overnight in a humidified chamber on a shaker at 4 °C. Slides were then washed three times (10 min each) with PBS-0.1% Tween-20 (Sangon Biotech, cat. no. A600560-0500), sections were stained with matched secondary antibodies (Alexa Fluor 647 Donkey anti-mouse, Jackson Immuno Research, 715-605-151; Alexa Fluor 568 Donkey anti-rabbit, Thermo Fisher Scientific, A10042; Alexa Fluor 647 Donkey anti-rabbit, Jackson Immuno Research, 711-605-152) at room temperature for 2 h. Staining with secondary antibodies alone was used as the control. The slides were washed 6 times (10 min each) with PBS-0.1% Tween-20, and a final wash was performed with PBS. Slides were mounted by adding 20 μL Antifade Mountain containing 4′,6-diamidino-2-phenylindole, dihydrochloride (DAPI) (Beyotime, cat. no. P0126-25) to each tissue. Then a coverslip was carefully placed on top of the slides. Fluorescence images were acquired using a confocal microscope (Olympus FV4000). All the images were further processed with OlyVia software.

### Comparison of T cell genes and signatures between primary and metastatic tumors in patient cohorts

We integrated RNA-sequencing (RNA-seq) data from TCGA-LUAD (primary) and META-PRISM (lung metastases) to compare T cell landscapes. Both datasets were processed via Kallisto/TxImport and log-transformed.^17^^,^^18^ To remove technical artifacts—including library size, batch effects, and tumor purity (inferred via ESTIMATE^19^)—we normalized the merged data using RUV–III–PRPS.^20^ This approach utilized pseudo-replicates and negative control genes to isolate biological variance. Finally, we evaluated eight T cell state and recruitment signatures using gene set variation analysis (GSVA)^21^ and visualized the mean scores across groups (Table S3).^22^

### Establishment of mouse lung tumor models

The establishment of mouse lung tumor models was based on established methods outlined in the previous work.^23^ Specifically, two-month-old *Kras*^G12D^;*Trp53*-deficient (KP) mice (B6-KP, GemPharmatech, Strain No. T015831) were chosen and subjected to anesthetic treatment. Then, adenoviral vectors expressing Cre, or empty vector controls (Obio Technology Company, Shanghai, China), were intratracheally delivered into the mouse lungs. Each mouse received a dosage of 2.5 × 10^7^ viral genome copies diluted in warm 50 μL sterile saline. After treatment, mice were kept on a warm pad until they recovered, with lung tissues collected at different time points (0, 4, 8, and 12 weeks) post-viral delivery for single-cell sequencing (Table S2).

Spontaneous breast cancer lung metastasis mouse model, MMTV-PyMT mice (FVB-MMTV-PyMT, GemPharmatech, Strain No. T004993) were established using female mice obtained from GemPharmatech, known to develop mammary gland carcinoma and progressive lung metastasis from 6 weeks of age onwards. Following housing in a controlled environment, lung tissues were collected at different time points (6-, 8-, 12-, 16-week age) for single-cell sequencing. In addition, breast tissues were harvested at 12 and 16 weeks from two mice whose corresponding lung tissues were included in the analysis, allowing for matched inter-organ comparison (Table S2).

To establish melanoma lung metastasis models, B16-F10 cells (B16-F10, ATCC, reference CRL-6475) were cultured and prepared for mouse modeling (mouse: C57BL/6, Charles River Laboratories, reference C57BL/6NCrl). After two passages in a cell culture incubator, cells were injected into two-month-old female mice via the tail vein. Each mouse received a slow intravenous injection of 100,000 cells in a total volume of 100 μL. Subsequently, lung tissues were collected at different time points (0, 1, and 4 weeks) after the above injection for single-cell sequencing.

All animals were housed in specific pathogen-free conditions at the Peking University animal facility, adhering to ethical regulations. All animals were experimentally naive, and all procedures were conducted in accordance with national guidelines for laboratory animal housing and care, as well as institutional regulations reviewed and approved by the Institutional Animal Care and Use Committee at Peking University.

### Histology of mouse lung tissues

Mice were anesthetized, and the right heart was perfused with PBS. Lungs were inflated with 4% paraformaldehyde and were continually fixed in 4% paraformaldehyde at 4 °C for 24 h. Then, the lungs were cryoprotected in 30% sucrose and embedded in optimal cutting temperature (Tissue Tek). Hematoxylin–eosin staining was performed as detailed above. For immunofluorescence, the lung tissues were cryosectioned at 15 μm thickness and air-dried overnight at room temperature. After removing the optimal cutting temperature, sections were blocked and permeabilized with 3% bovine serum albumin plus 0.1% Triton X-100 in PBS for 1 h, and then incubated at 4 °C overnight with primary antibodies (rabbit anti-CD8a, Abcam, ab217344; mouse anti-PD-1, Servicebio, GB12338; rat anti-Ly6G, Biolegend, 127602; rabbit anti-STEAP4, Proteintech, 11944-1-AP). After washing, sections were stained with matched secondary antibodies (Alexa Fluor 647 Donkey anti-mouse, Jackson Immuno Research, 715-605-151; Alexa Fluor 568 Donkey anti-rabbit, Thermo Fisher Scientific, reference A10042; Alexa Fluor 647 Donkey anti-rat, Jackson Immuno Research, reference 712–605153; Alexa Fluor 647 Donkey anti-rabbit, Jackson Immuno Research, 711-605-152 at room temperature for 2 h. Images were captured with a confocal fluorescence microscope (Olympus, FV4000).

### Single-cell collection, library preparation, and sequencing for mouse samples

After deep anesthesia, mice at respective time points underwent subsequent procedures. Specifically, to eliminate blood cells from the lungs, mice were perfused with PBS through their left ventricles. Subsequently, a 1 mL enzyme solution comprising 5 U/mL neutral protease (Worthington-Biochem, LS02111) and 0.33 U/mL DNase I (Roche, 10104159001) was instilled into the lungs via the trachea. The lungs filled with the enzyme solution were further immersed in the same solution at room temperature for 45 min. Following enzymatic incubation, the digested lung tissues were gently minced into small pieces and incubated in Dulbecco's modified Eagle medium 1640 (Invitrogen) containing 10% fetal bovine serum at room temperature for 10 min. A single-cell suspension was obtained by passing the cells through 100 μm and then 40 μm cell strainers. The cell suspension was subsequently centrifuged at 800 rpm for 10 min to form a pellet. This pellet was resuspended in an RBC lysis buffer and incubated at room temperature for 10 minu to eliminate red blood cells. After centrifugation at 800 rpm for 10 min, the supernatant was removed.

Next, cells were resuspended in FACS staining buffer to a final volume of 1 mL, and anti-CD45 antibody (PE/Cyanine7 anti-mouse CD45 Antibody, BioLegend, 103114) was added at a ratio of 1:500. The cells were gently agitated every 10 min to prevent cell clumping and settling. After 30 min, unbound antibodies were removed using FACS staining buffer, and the centrifuged cells were resuspended in FACS buffer before DAPI staining. CD45^+^ cells were sorted using the single-cell select mode in BD FACS Aria II and III instruments.

Subsequently, an equal number of CD45^+^ and CD45^–^ cells were mixed in a 1:1 ratio and processed following the 10X genomics protocol. Briefly, sorted cell subsets were loaded onto the 10X Chromium system and encapsulated using the Single Cell 50 Library & Gel Bead Kit (Chromium Single Cell 50 Library and Bead Kit, 10X Genomics, cat. no. 1000006; Chromium Single Cell 50 Library Construction Kit, 10X Genomics, cat. no. 1000020). Single-cell gene expression profiles were generated according to the manufacturer's instructions. The completed libraries were sequenced on HiSeq4000 Illumina platforms with a targeted median read depth of 50,000 reads per cell from total gene expression libraries.

### Neutrophil isolation from human whole-blood samples

Fresh whole-blood samples were collected from human donors. Peripheral blood was slowly layered onto Polymorphprep (Polymorphprep, No. 1895) without mixing the blood into the lower Polymorphprep layer. Centrifugation was performed at 500 *g* at 20 °C for 35 min with the lowest acceleration/deceleration settings. The neutrophil layer (polymorphonuclear leukocytes) was carefully aspirated using a pipette, avoiding contamination from the upper mononuclear cell layer. Cells were washed with 5 mL of 50% Hanks' balanced salt solution (HBSS) (Gibco, 24020117) and resuspended by gentle pipetting. Subsequent centrifugation steps included: 350 *g* at 20 °C for 10 min, followed by supernatant removal (avoiding cell loss by not tilting the tube). Cells were resuspended in 10 mL HBSS, centrifuged at 300 *g* for 5 min at 20 °C, and treated with RBC lysis buffer (Invitrogen, 00-4333-57). After lysis, cells were centrifuged again (300 *g*, 5 min, 20 °C), washed with 10 mL HBSS, and pelleted at 300 *g* at 20 °C for 5 min. The final pellet contained purified neutrophils.

### Flow cytometry

Isolated neutrophils were resuspended in PBS containing 2% fetal bovine serum and then incubated with FcR blocking reagent human (BD, 564220) for 15 min. Cells were incubated with fluorochrome-conjugated antibody mix (BD Horizon™ BV421 Mouse Anti-Human CD15, BD, 567008; Alexa Fluor® 647-conjugated Anti-Human STEAP4, R&D Systems, FAB4626R; BD Pharmingen™ PE Mouse Anti-Human CD16, BD, 561313) at 4 °C in the dark for 30 min. Cell suspension was washed twice and stained with eBioscience™ 7-AAD Viability Staining Solution (eBioscience, 00-6993-50) and analyzed on a BD FACS Aria SORP. Data processing was performed using FlowJo™ v10 software (FlowJo LLC).

### Immunostaining of whole-blood samples

Whole-blood samples from normal mice and MMTV-PyMT (early, mid, late) mice were collected in heparin-containing collection tubes. After two rounds of red blood cell lysis with 1 × RBC lysis buffer (Invitrogen, 00-4333-57) for 5 min and washed with working buffer (5% fetal bovine serum plus PBS), the single-cell suspensions of whole blood cells were fixed with 2% paraformaldehyde for 10 min. The cell suspension was dropped onto glass slides and air-dried overnight. Then, the single cells were ready for the following staining procedure. The single cells were permeabilized (0.1% Triton X-100, 0.1 M Glycine, PBS) for 10 min. Slides were washed and blocked with blocking buffer (3% BSA + 0.1% Tween-20 + 10% normal donkey serum) for 1 h. Incubations with primary antibodies were performed overnight at 4 °C. Incubation with secondary antibodies conjugated to Alexa Fluor (Jackson Immuno Research) was performed for 1.5 h. After several washes with PBS, sections were stained with DAPI at room temperature for 5 min, rinsed with water, and mounted with PermaFluor mounting medium (Beyotime).

For human samples, the isolated neutrophils were fixed in 2% paraformaldehyde, and smears were prepared for immunofluorescence analysis. The slides were washed and blocked with blocking buffer (3% BSA + 0.1% Tween-20 + 10% normal donkey serum) for 1 h. Slides were then stained with primary antibodies (mouse anti-CD15, Abcam, ab241552; rabbit anti-STEAP4, Proteintech, 11944-1-AP) at 4 °C overnight. Next, secondary antibodies conjugated to Alexa Fluor (Jackson Immuno Research) were used for 1.5 h. After several washes with PBS-Tween 20, sections were stained with DAPI at room temperature for 5 min, rinsed with water, and mounted with PermaFluor mounting medium (Beyotime). Fluorescence images were acquired using a confocal microscope (Olympus FV4000). All the images were further processed with OlyVia software.

### scRNA-seq data processing and unsupervised clustering

Single-cell RNA-sequencing (scRNA-seq) data were aligned and quantified using the CellRanger toolkit v7.1.0 against the reference genome mm10. Preliminary filtered data generated from Cell Ranger were then imported into the R-based package, Seurat v4.3.0, for downstream analysis.^24^ The high-quality cells were kept if they satisfied the two metrics: i) ≥200 expressed genes, ≥800 UMIs, and mitochondrial UMIs ≤30%; ii) ≤6000 expressed genes and ≤40000 UMIs. Scrublet was then applied to remove potential doublets with an expected doublet rate of 6%, and cells with more than 0.95 quantiles of doublet score were removed in the downstream analysis.^25^ The raw count data were normalized using a scale factor of 10^4^ by the NormalizeData function. The top 2000 highly variable genes were selected by *FindVariableFeatures* (selection.method = "vst"). The scaled gene expression levels of these genes were used for principal component analysis for dimensionality reduction using the *RunPCA* function with parameter "npcs = 30". To remove batch effects between samples, we performed the Harmony algorithm to generate batch-corrected principal components using the *RunHarmony* function with group.by.vars = "sample". Next, the top 20 principal components were used for graph-based clustering by the *FindNeighbors* and *FindClusters* functions. All cells were projected to a two-dimensional plot for visualization based on the Uniform Manifold Approximation and Projection (UMAP) method implemented by the *RunUMAP* function using the top 20 PCs.

### Cell cluster annotation

The major cell types were annotated based on the first-round clustering according to well-known marker genes: *Epcam*, *Krt19*, and *Cdh1* for epithelial cells; *Pecam* and *Cldn5* for endothelial cells; and *Col1a1* and *Msln* for stromal cells. The immune cells were marked by *Ptprc*, and then clustered based on Cd3d, Cd3e, Cd4, and Cd8a for T cells; Nkg7 and Ncr1 for natural killer cells; Cd79a for B cells; and Ly6c2, Fcer1g, Cd68, Marco, Csf1r, Csf3r, Cd209a, Lamp3, Xcr1, Fscn1, and S100a8 for myeloid lineages. Next, the second-round unsupervised clustering was based on each major cell type (T cells, natural killer cells, myeloid cells, epithelial cells, endothelial cells, stromal cells) by the same procedure as the first-round clustering. Different resolution parameters for unsupervised clustering were examined. The markable genes of each cell cluster were identified by the *FindAllMarkers* function in Seurat. Cell types were manually annotated according to the markers. Neutrophil marker genes in Figure 3 are listed in Table S6.

### Identification of the maturity of neutrophils

To predict the cell differentiation order among neutrophil cell states, neutrotime signature genes published by Grieshaber-Bouyer and colleagues were applied using calculate signature score.^26^ N1 cluster was highly expressed in immature neutrophils, as shown by the markers such as *Camp*, *Ngp*, and *Ltf*, and exhibited the earliest Neutrotime.^27^ Therefore, the N1 cluster was identified as immature neutrophils and chosen as the initiated state in the following trajectory analysis.

### Trajectory analysis of neutrophil subsets

To predict the cell differentiation trajectory, we applied RNA velocity and Slingshot in Figure 2G. RNA velocity was performed by the following steps: First, the spliced and unspliced UMIs were distinguished by the Python-based velocyto tool. Next, the data were imported into the scVelo package to estimate RNA velocities. After filtering the genes and normalizing the data by the scvelo.pp.filter_and_normalize function with default parameters, the moments were calculated by scvelo.pp.moments. The generalized dynamical model was used to estimate RNA velocity using the function scvelo.tl.recover_dynamics, scv.tl.velocity(mode = "dynamical"), and scv.tl.velocity_graph. Finally, the velocities were projected onto the UMAP embedding using scv.pl.velocity_embedding_stream function for visualization (Fig. 3G). Slingshot R package (version 2.0.0) was used to identify the neutrophil lineage structures. Slingshot was implemented in the analysis after dimensionality reduction and clustering of the integrated neutrophil data. UMAP embedding and cell cluster labels served as an input to the function slingshot (reducedDim, clusterlabels, start.clus = "N1", extend = "n", stretch = 0, omega = FALSE). The global lineage structure was identified with a cluster-based minimum spanning tree (MST), and then principal curves were fitted to model the development of cells along each lineage.

**Figure 1:**
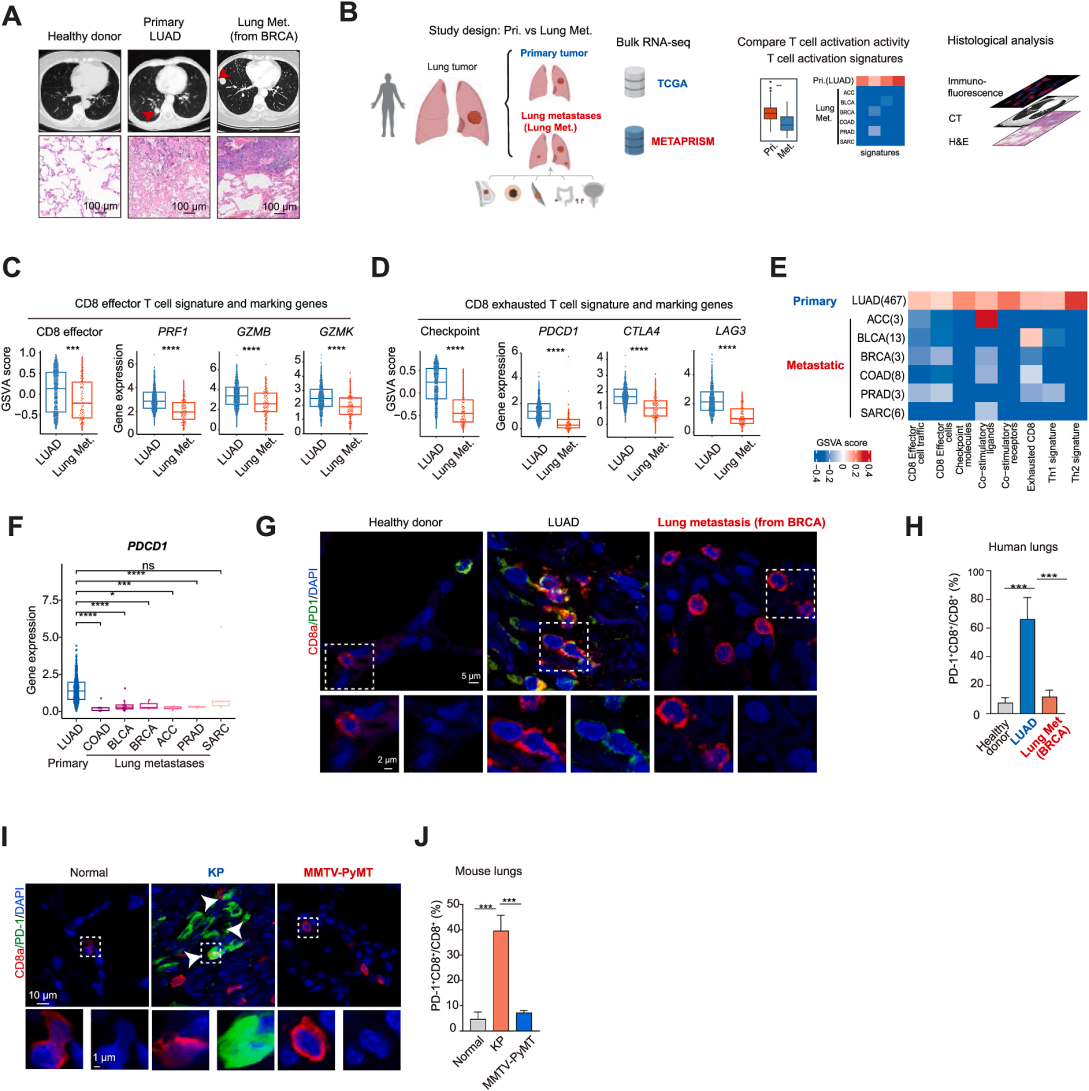
Lung metastases from various origins establish a T cell-suppressed tumor microenvironment. **(A)** Representative chest computed tomography images taken at the level of the carina. Hematoxylin–eosin histological staining of the lungs of a healthy donor, lung adenocarcinoma (LUAD) patient, and breast cancer (BRCA) patient. **(B)** Study design of identifying the characteristics of the lung metastatic tumor microenvironment using the primary lung tumor as a reference. In humans, comparison of T cell activation based on RNA-seq data of TCGA and METAPRISM cohorts, and validation using histological analysis. Lung Met., lung metastases. **(C, D)** Box plots comparing batch-corrected gene expression or signature score of T cell effector (C), and checkpoint molecule signature (D) between lung primary tumors (LUAD, *n* = 436) and lung metastases (*n* = 100). **(E)** Heatmap of the normalized activity of T cell signatures across lung tumors from different cancer types. Lung metastatic tumors were classified by their originating primary cancer types. Sample numbers were noted in brackets. **(F)** Box plots showing *PDCD1* expression level in lung primary and metastatic tumors. **(G)** Typical images of immunostaining using antibodies against CD8a and PD-1 of lungs from LUAD and BRCA patients. Scale bar, 5 μm or 2 μm. **(H)** The percentages of CD8^+^PD-1^+^ T cells among CD8^+^ T cells in human donors are shown in (G). Data were presented as mean ± standard error of the mean (*n* = 3). ∗∗∗*p* < 0.001 by Student's *t*-test. **(I, J)** Representative immunofluorescence images for CD8a (red) and PD-1 (green) in mouse lungs from normal, primary lung tumor (KP), and lung metastasis (MMTV-PyMT) models. **(I)** Nuclei were counterstained with DAPI (blue). The white arrowheads indicate CD8^+^PD-1^+^ T cells, and the dashed boxes outline the magnified regions shown in the bottom panels. **(J)** Quantification of the percentages of PD-1^+^ cells among CD8^+^ T cells in the lungs. Data were presented as mean ± standard error of the mean (*n* = 3). ∗∗∗*p* < 0.001 by Student's *t*-test. Scale bar: 10 μm or 1 μm.

**Figure 2:**
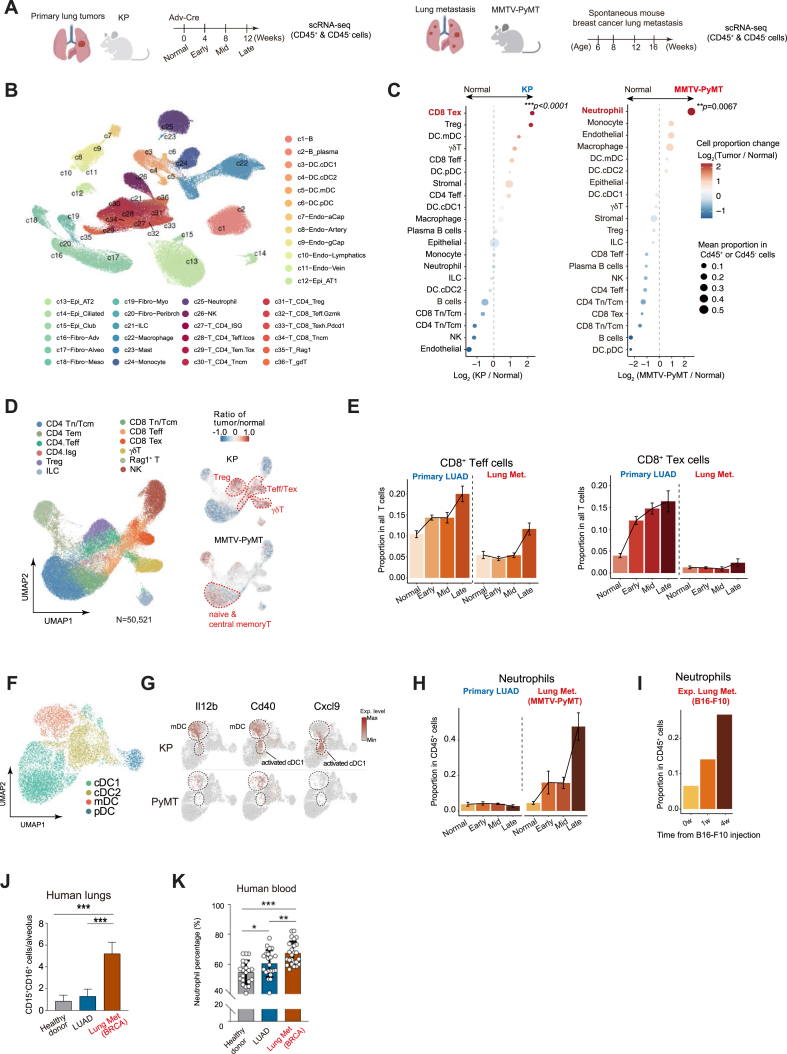
A dynamic single-cell atlas of microenvironmental remodeling in primary lung tumors and lung metastases. **(A**) Experimental design for single-cell RNA-seq workflow. CD45^+^ and CD45^–^ cells were sorted from the mouse lungs of MMTV-PyMT and KP models at four time points for each condition. **(B)** Uniform manifold approximation and projection (UMAP) plots showing the annotated cell subsets of the single-cell atlas obtained from mouse models shown in (A). **(C)** Comparison of cell type abundance between primary (left) or metastatic (right) tumor microenvironment and normal lung microenvironment. Cell proportions in CD45^+^ or CD45^–^ cells for each cell type in each sample were used for comparison. Student's *t*-test. **(D)** UMAP plot showing T cell subsets in KP and MMTV-PyMT mice. Mouse number of each time point in the KP model: 4, 3, 3, and 3 in the MMTV-PyMT model: 3, 6, 3, and 3 (Table S2). Relative kernel densities showing cell enrichment (red) and depletion (blue) in UMAP space for pairwise comparisons of tumor versus normal controls. **(E)** Mean proportions of CD8^+^ Teff and Tex cells in KP and MMTV-PyMT mouse lungs. **(F)** UMAP plot of dendritic cell (DC) subpopulations in KP and MMTV-PyMT mouse lungs. cDC1, conventional type 1 DC; cDC2, conventional type 2 DC; mDC, mature Ccr7^+^ DC; pDC, plasmacytoid DC. **(G)** UMAP plots showing expression level (log_2_(TPM)) of selected genes in KP and PyMT. **(H, I)** Bar plot showing mean neutrophil proportion in CD45^+^ cells across samples of each time point of KP and MMTV-PyMT models (H) (mean ± standard error of the mean) and B16-F10 experimental lung metastasis model (I). **(J)** The CD15^+^CD16^+^ neutrophils in the alveolus were quantified based on immunofluorescence staining in the lungs from the healthy donors, lung adenocarcinoma patients, and breast cancer patients, as shown in Figure S2G. Data were expressed as mean ± standard error of the mean. ∗∗∗*p* < 0.001 by Student's *t*-test. **(K)** The proportion of neutrophils in whole blood from healthy donors, lung adenocarcinoma patients, and breast cancer patients (mean ± standard error of the mean, *n* ≥ 20).

**Figure 3:**
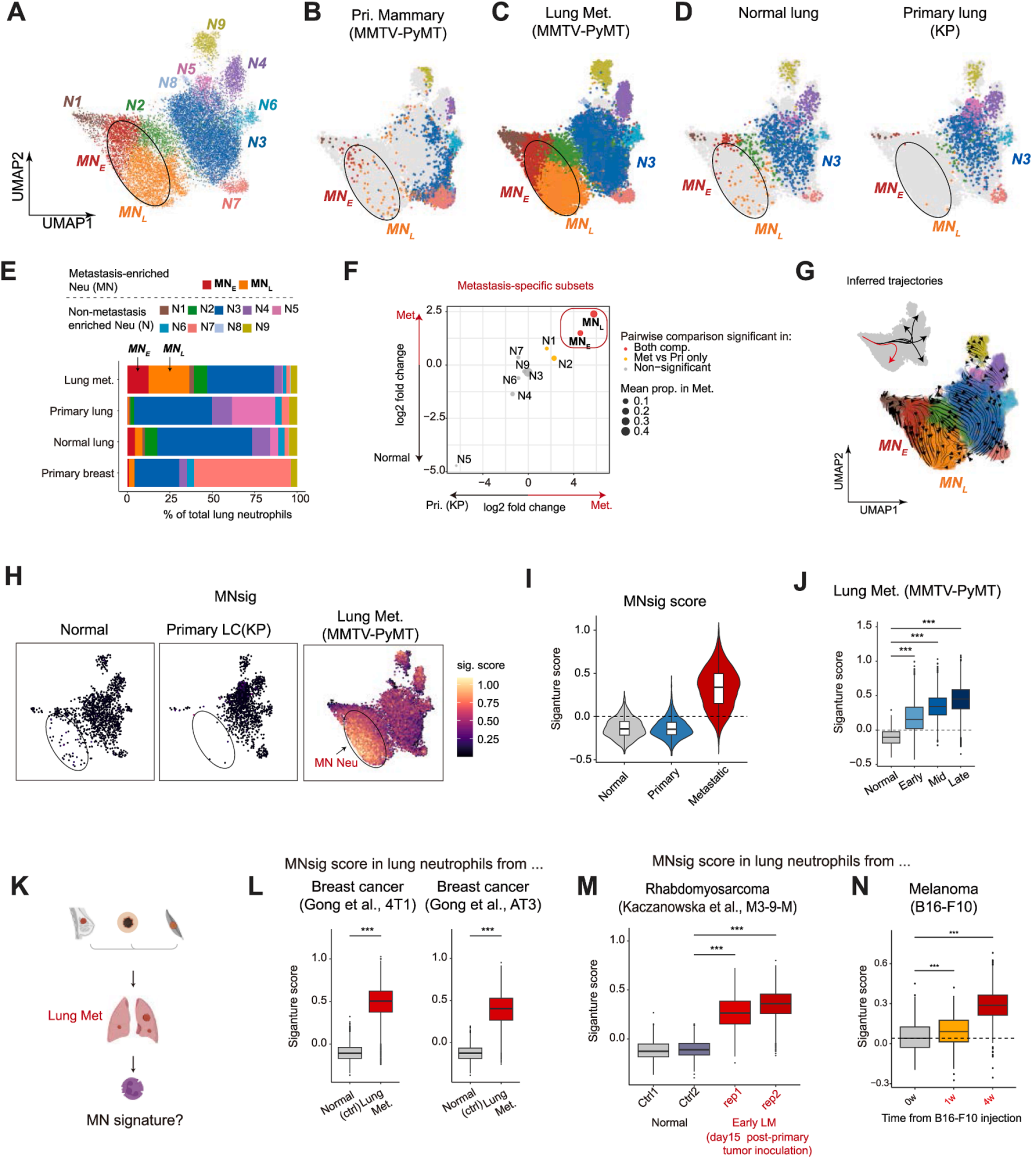
Neutrophils in the lung metastatic niche share a metastasis-specific gene signature across diverse tumor types. **(A)** Uniform manifold approximation and projection (UMAP) plots showing the neutrophil subsets integrated from (B–D). **(B, C)** UMAP plots showing neutrophil subsets in the integrated data of lungs from the primary sites and metastatic sites of the MMTV-PyMT model. Primary mammary tumor samples are from 12- (mid) and 16-week (late)-aged mice. **(D)** UMAP plots showing neutrophil subsets in the integrated data of lungs from normal lungs and primary lung tumor from the KP model. **(E)** Bar plot showing the proportion of each neutrophil subset within total neutrophils in each condition. **(F)** Comparative analysis of neutrophil subset proportions in lung metastasis (Met.) versus normal lungs, and lung metastases in MMTV-PyMT versus primary lung tumors (Pri.) in the KP model. Statistical significance (*p* < 0.05) was determined by the Wilcoxon rank-sum test. **(G)** UMAP plots showing the direction of differentiation between neutrophil subsets inferred by RNA velocity and the Slingshot algorithm (left top). **(H)** UMAP plots showing the expression level of MNsig in neutrophils from normal, primary tumor-bearing (from KP), and metastatic lungs (from MMTV-PyMT). **(I)** Violin plot quantifying the distribution of MNsig scores in neutrophils across the three conditions, corresponding to the UMAP visualization in (H). **(J)** Box plot showing the changes in score of MNsig signature genes (65 genes) across time within MN4 neutrophils from lung metastases in MMTV-PyMT. ∗∗∗*p* < 0.001 by Student's *t*-test. **(K)** Schematic of the analyses (L–N) designed to test for the induction of the neutrophil MNsig in lung metastases from various tumor models. **(L)** Comparison of MNsig levels in neutrophils from the normal lung and the metastatic niche, based on scRNA-seq data from.^30^ The analysis used two orthotopic breast cancer mouse models, 4T1 and AT3. **(M)** Comparison of MNsig levels in neutrophils from the normal lung and the metastatic niche, based on scRNA-seq data.^31^ This analysis used an orthotopic rhabdomyosarcoma mouse model at a pre-metastatic stage (day 15 post-primary tumor inoculation). **(N)** Box plots comparing MNsig levels in neutrophils from normal lungs versus the metastatic niche, using scRNA-seq data from a B16-F10 experimental metastasis model created by tail vein injection.

### Identification of the MNsig signature genes

The MNsig neutrophil signature genes were obtained from the differentially expressed genes in the MN_L_ subset versus other subsets using the following threshold: log_2_ fold change > 0.4 and adjusted *p*-value < 0.05 in the MN_L_. We obtained 65 genes used as MNsig (Table S4). Signature score for each cell of the integrated data across all conditions was calculated by the *AddModuleScore* function in Seurat with default parameters, which estimates module activity based on average gene expression (Fig. 3). The identification of enriched Gene Ontology terms of MNsig genes was employed by *clusterProfiler*^28^ and visualized by the R package *GOplot* using a chord diagram.^29^

### Identification of the highly expressed membrane protein in the MN neutrophil subset

The Gene Ontology "plasma membrane" GO:0005886 was used to select membrane protein-coding genes. The MN_L_ up-regulated genes compared with other neutrophil subsets (adjusted *p*-value < 0.05, expression percentage > 10%, and log_2_ fold change > 0.5, calculated from the *FindAllMarkers* function described above) were used to select membrane proteins. Among these proteins (*Steap4*, *Kit*, *Mif*, *Plscr1*, *Ltb4r1*, *Mcam*, and *Vim*), *Steap4* had the highest expression percentage (55%), exhibiting specificity to lung metastasis.

### Screening for candidate membrane protein specific to lung metastasis

For each sample, we calculated the expression percentage of genes in the MNsig. The samples were stratified into three groups: neutrophils from normal lung tissues, neutrophils from primary lung cancer in the KP model, and neutrophils from lung metastases in the MMTV-PyMT model. For each gene, we calculated the mean expression percentage within each group and determined the log_2_ fold changes (log_2_FC) when comparing MMTV-PyMT with either the normal or KP groups. To address extreme log_2_FC values resulting from small expression percentages, we introduced a "proportion-weighted log_2_FC" metric. This metric combined the mean expression percentage in the lung metastasis group with log_2_FC, providing a balanced measure that accounts for both the absolute value of expression percentage and the relative changes across comparisons. The proportion-weighted log_2_FC ensured the selection of genes with both high expression percentages and strong specificity for lung metastasis.

### Prediction of lung metastasis using membrane protein genes on neutrophils based on scRNA-seq data

To evaluate the performance of STEAP4 and other known membrane proteins in predicting lung metastasis, we utilized the expression percentage of these proteins on neutrophils as indicators, based on scRNA-seq data. Using expression percentages, not gene expression values, could help mitigate biases that arise from batch effects across different datasets. The validation dataset included lung metastasis samples from our B16-F10 experimental metastasis model, samples from 4T1 and AT3 models from Gong and colleagues,^30^ and samples from the M3-9-M model from Kaczanowska and colleagues.^31^ The control samples were integrated from neutrophils in the normal lungs from our data and the above-mentioned published data. For each sample, the expression percentage of each gene in neutrophils was calculated by determining the frequency of non-zero expressing cells among neutrophils identified through scRNA-seq profiles. ROC analysis was conducted using the R package pROC, with the roc function for plotting ROC curves and the auc function for calculating the area under the curve (AUC).

### Statistical analysis

Unless otherwise mentioned, the data were depicted as mean ± standard error of the mean (as denoted in the figure legends). The figures showcase data gathered from numerous independent experiments, which were conducted on separate occasions using distinct samples. By default, the data displayed within the figure panels were derived from a minimum of three separate experiments. To assess the statistical significance of the mean differences between samples, two-tailed Student's *t*-tests were employed (∗*p* < 0.05, ^∗∗^*p* < 0.01, and ^∗∗∗^*p* < 0.001).

## Results

### Lung metastases from various origins establish a T cell-suppressed TME

We first observed distinct morphological characteristics of the lung metastatic lesions compared with the primary lung tumor, indicating unique TME specific to lung metastasis (Fig. 1A). Given the pivotal role of T cells in anti-tumor immunity and their association with immunotherapy efficacy,^33^ we investigated the immune phenotypes of primary and metastatic TMEs. RNA-seq data of LUAD tumors from TCGA and lung metastases from the META-PRISM cohort were analyzed (Fig. 1B; Methods).^18^^,^^34^ Comparative transcriptional profiling revealed that T cell activation signatures were significantly down-regulated in lung metastases compared with primary lung tumors (Fig. 1C; Fig. S1A). Most T cell activation-associated signatures, including effector CD8^+^ T cell markers such as *PRF1*, *GZMB*, and *GZMK*, exhibited reduced expression in lung metastases (Fig. 1C; Fig. S1A).^35^ Correspondingly, immune checkpoint molecules *PDCD1*, *CTLA4*, and *LAG3*, typically up-regulated during sustained T cell activation, were also expressed at lower levels in lung metastases (Fig. 1D). These observations suggest that T cells in lung metastases exhibit reduced activation towards effector or exhausted states.

To further explore whether these reductions were influenced by the primary tumor origin, lung metastases were stratified based on their primary tumor types, and we observed that metastatic tumors from multiple different primary origins consistently displayed lower expression level of T cell activation signatures and effector genes compared with LUAD (Fig. 1E; Fig. S1B). Using PD1 as a surrogate marker of T cell activation,^36^ we observed a significant reduction in both *PDCD1* RNA expression in colon cancer, breast cancer, bladder cancer, renal cancer, and stomach cancer (Fig. 1F). These results suggest that lung metastases, regardless of their primary tumor origins, establish an immunosuppressive environment.

To further validate the immune microenvironment characteristics of lung metastasis, we compared the proportion of PD1-expressing CD8^+^ T cells across healthy human lung tissues, primary lung cancer, and breast cancer lung metastases (Cohort 1; Table S1). Consistent with our transcriptional data, the frequency of activated CD8^+^ T cells in lung metastases was markedly lower than that in primary lung tumors (Fig. 1G and H). Consistently, similar phenomena were observed in syngeneic mouse models (Fig. 1I and J). These findings collectively indicate that the lung metastatic microenvironment, in both humans and mice, is characterized by a paucity of activated T cells. This deficiency likely represents a critical factor contributing to immunotherapy resistance, necessitating a thorough investigation into the underlying mechanisms driving this state.

### A dynamic single-cell atlas of microenvironmental remodeling in primary lung tumors and lung metastases

To uncover the mechanistic basis driving the distinct microenvironments of primary lung tumors and lung metastases, we mapped their evolutionary trajectories at single-cell resolution. We sought to track the stepwise phenotypic transformations of diverse cellular constituents as the lung milieu transitions from a steady-state physiological condition to a pathological landscape. To this end, we employed two murine models (Fig. 2A). For primary lung tumors, we utilized KP mice to model lung adenocarcinoma that recapitulates the TME dynamics of human disease^23^ (Fig. 2A). For lung metastasis, we leveraged the MMTV-PyMT model, wherein mammary tumors spontaneously metastasize to the lung, enabling longitudinal analysis of TME remodeling during metastatic colonization^37^^,^^38^ (Fig. 2A). By performing longitudinal scRNA-seq across normal, early-, middle-, and late-stage diseases in both models, we generated a comprehensive cross-disease lung tumor cell atlas encompassing 201,977 high-quality cells (Fig. 2B; Table S2; Methods). This robust dataset provides a granular blueprint of how the lung microenvironment is progressively remodeled in distinct disease contexts.

To delineate the microenvironmental evolution in both primary and metastatic contexts, we systematically annotated the diverse cellular populations based on their distinct transcriptomic profiles (Fig. 2B). We identified major lineages including T cells, B cells, macrophages, neutrophils, natural killer cells, endothelial cells, fibroblasts, and epithelial cells, alongside their corresponding subsets (Fig. 2B). Analysis of the immune cell compartment within this atlas revealed a striking dichotomy between primary and metastatic lung tumors (Fig. S2A and S2B). Primary KP lung tumors maintained a lymphocyte-dominated TME, characterized by a robust enrichment of cell types associated with T cell activation, notably exhausted CD8^+^ T cells (CD8^+^ Tex), effector CD8^+^ T cells (CD8^+^ Teff), and dendritic cells (Fig. 2B; Fig. S2A and S2B). In stark contrast, lung metastases exhibited progressive temporal evolution toward myeloid cell predominance, particularly neutrophils and monocytes (Fig. 2B; Fig. S2A and S2B).

To understand the organization of the TME during lung metastasis progression, we constructed a cellular correlation network, linking covarying cell populations based on their relative proportions within the immune and non-immune compartments across multiple time points. Network analysis revealed fundamentally distinct patterns of cellular organization between the primary and metastatic TMEs (Fig. S2C). For instance, in the primary TME, activated T cell states (CD4 Teff, CD8 Teff, and CD8 Tex) strongly correlated with dendritic cells (conventional type 1 dendritic cells and mature Ccr7^+^ dendritic cells), forming a cohesive network module indicative of a well-coordinated and active immune response (Fig. S2C). Strikingly, this coordinated immune module was completely absent in lung metastases.

Analysis of the dynamic changes in CD8^+^ T cell states further demonstrated that both effector and exhausted populations were enriched in primary lung tumors and progressively expanded during tumor progression (Fig. 2D and E; Fig. S1C). Conversely, T cell populations in the lung metastatic TME consistently remained dominated by inactive states throughout the entire disease course (Fig. 2D and E; Fig. S1C). Furthermore, given the critical role of dendritic cells in priming and activating T cell-mediated immunity, and their prominent identification within our cellular network (Fig. S2C), we evaluated the expression profiles of dendritic cell-derived activating factors. We found that key molecules essential for T cell activation and recruitment, such as *Il12b*, *Cd40*, and *Cxcl9*, were robustly expressed by dendritic cells within the primary lung TME (Fig. 2F and G). However, these crucial factors were notably deficient in the lung metastasis microenvironment (Fig. 2F and G). Furthermore, T cells in metastatic TME displayed reduced expression of interferon-g (IFN-g) (Fig. S1F), whereas myeloid cells and stromal cells failed to up-regulate the interferon response (Fig. S1F). This lack of essential costimulatory and chemokine signals further corroborates that T cells in lung metastases are deprived of adequate activation cues, thereby being locked in an immunosuppressive state. Importantly, these molecular expression profiles strongly validate the biological robustness of the distinct cellular modules identified in our network analysis.

To determine whether T cell suppression is a conserved hallmark of lung metastases across diverse cancer types, we profiled the B16-F10 experimental melanoma metastasis model. By performing temporal scRNA-seq on lung tissues harvested at 0, 1, and 4 weeks post-intravenous injection, we captured the dynamic immune landscape during metastatic colonization (Methods). We then integrated these temporal datasets, alongside our MMTV-PyMT data, with a publicly available scRNA-seq cohort of the M3-9-M rhabdomyosarcoma lung metastasis model.^31^ Strikingly, this comprehensive cross-model analysis revealed a uniform and profound deficiency in effector molecule signatures within both CD8^+^ and CD4^+^ T cell compartments across all three distinct metastatic models, a suppressed state that persisted continuously throughout all stages of disease progression (Fig. S1D and S1E). Overall, these results indicate that lung metastases establish a robust and consistent immunosuppressive microenvironment—characterized by an abundance of inactive T cells—regardless of the primary tumor's origin, highly consistent with our observations in human patients.

### Neutrophil accumulation is closely related to lung metastases

Notably, by leveraging our dynamic single-cell atlas, we identified a striking shift within the myeloid compartment of the lung metastatic TME (Fig. S2A and S2B). Compared with other myeloid populations such as monocytes and macrophages, neutrophils exhibited the most dramatic and rapid expansion during metastasis progression (Fig. S2D). They accumulated substantially from the initial stages of disease and ultimately became the predominant immune cell type within the late-stage metastatic niche in the MMTV-PyMT model (Fig. 2H). Furthermore, the B16-F10 experimental lung metastasis model corroborated this progressive neutrophilic influx during disease progression (Fig. 2I). Consistently, immunofluorescence staining in the MMTV-PyMT mouse model confirmed a robust increase in neutrophil infiltration within lung metastases relative to both normal lung controls and primary lung tumors (Fig. S2E and S2F).

To further validate this robust neutrophil accumulation and its clinical relevance, we assessed infiltration of CD15^+^CD16^+^ neutrophils via immunofluorescence staining in human cohorts. In patients with breast cancer lung metastases, neutrophils were significantly enriched within the metastatic lesions compared with normal lung tissues from healthy donors and primary LUAD tumors (Fig. 2J; Fig. S2G). Moreover, analysis of human peripheral blood revealed a significant increase in circulating neutrophils in breast cancer patients with lung metastasis, compared with the patients with primary lung tumors alone (Fig. 2K).

Collectively, the profound accumulation of neutrophils in lung metastasis highlights their critical role in shaping the pathological metastatic microenvironment. Given that neutrophils frequently acquire myeloid-derived suppressor cell characteristics with potent immunosuppressive functions,^10^ their massive expansion likely represents a primary mechanism underlying the severe T cell suppression observed in lung metastases.

### Identification of a metastasis-specific gene signature in neutrophils within the metastatic niche

Neutrophils play important roles in both primary and metastatic lesions.^39^, ^40^, ^41^, ^42^, ^43^ However, whether neutrophils exhibit functional heterogeneity in primary versus metastatic settings was understudied. To identify neutrophil states unique to the metastatic lung microenvironment, our primary investigation focused on comparing neutrophils from primary lung tumors (KP model) with those from lung metastases (MMTV-PyMT model), thereby controlling for the organ niche, which was found to strongly influence neutrophil phenotypes.^14^ However, a potential confounder in this approach is the different tumors in the lung microenvironment. To address this, neutrophils from primary mammary tumors (Pri.Mammary) served as a control for comparison with their corresponding lung metastases within the same MMTV-PyMT model (Methods; Table S2). This intra-model analysis revealed markedly greater neutrophil heterogeneity in the metastatic site (Fig. 3A–C), confirming that the metastatic niche itself induces distinct neutrophil phenotypes, even within the same tumor genetic lineage. Furthermore, we observed that neutrophils in the MMTV-PyMT lung metastatic niche shared common subsets with those from normal lungs and KP primary tumors (*e.g.*, the N3 subset), which were notably absent in the primary mammary tumor (Fig. 3C and D). This may be because the lung tissue microenvironment exerts a terminal shaping effect on neutrophil phenotypes once they are recruited to the lung.^14^^,^^15^^,^^30^ Subsequently, we employed the neutrophils from normal lungs and primary lung tumors as controls to exclude the lung-specific signature and isolate the metastasis-specific phenotypes in our subsequent analyses.

Strikingly, we discovered two major populations exclusively enriched in the metastatic niche, metastasis-enriched neutrophil early (MN_E_), and metastasis-enriched neutrophil late (MN_L_) (Fig. 3A, C–F; Fig. S3A). The MN_E_ and MN_L_ populations were virtually absent in all non-metastatic sites we examined: the primary MMTV-PyMT breast tumors, the primary KP lung tumors, and normal lung tissue (Fig. 3A–F). Furthermore, the proportions of both populations progressively increased with metastatic advancement, while remaining absent throughout primary lung tumorigenesis (Fig. S3B). Taken together, these results indicate that the MN_E_ and MN_L_ populations are specifically induced by the metastatic context, rather than by the primary breast tumor or the lung organ niche.

To investigate the developmental relationship between MN_E_ and MN_L_, we performed a comprehensive analysis of their maturation and trajectory (Methods). A convergence of computational approaches confirmed a clear progression: RNA velocity analysis indicated a directional flow from MN_E_ to MN_L_ (Fig. 3G), while trajectory inference with Slingshot modeled this as a distinct, metastasis-specific lineage (red, Fig. 3G). This differentiation progression was further supported by the "Neutrotime" score, which positioned MN_E_ as an earlier subset, while MN_L_ represented a more mature, differentiated state (Fig. S3C). Collectively, these findings establish a clear MN_E_-to-MN_L_ differentiation trajectory that is unique to the metastatic niche.

The gene expression profiles of MN_E_ and MN_L_ were consistent with this differentiation axis and revealed a key distinction in their specificity. Consistent with their position as an intermediate state, MN_E_ expressed genes associated with early neutrophil maturation, such as *Prok2*, *Mmp8*, *S100a6*, and *Cd177* (Fig. S3A). These markers, however, were not exclusive to the metastatic niche and were also detected in the early neutrophil populations within normal lungs and primary lung tumors (*e.g.*, N1 and N2) (Fig. S3A), suggesting that they include a general maturation gene program.

In contrast, the MN_L_ subset down-regulated these early maturation genes (*e*.*g.*, *Cd177*, *Prok2*) and highly expressed genes (*e.g.*, *Steap4*, *Mif*, *Cstdc6*, *Stfa2*), which were specifically enriched throughout the metastatic states but largely absent in non-metastatic contexts (Fig. S3A). We reasoned that, as the stable, terminally differentiated endpoint of the MN_E_-to-MN_L_ trajectory, the gene program of the MN_L_ state would serve as the most robust representation of this metastasis-specific neutrophil identity. To validate this, we identify a gene signature from the top MN_L_ marker genes (Methods; Table S4), and observed that the signature was highly specific to the lung metastatic niche, with negligible expression in normal tissues or primary tumors (Fig. 3H and I; Fig. S3D). Furthermore, differential expression analysis confirmed that this gene signature was robustly up-regulated in the metastasis-specific populations (MN_E_ and MN_L_) relative to neutrophils from either primary lung tumors or normal lung tissue, highlighting its exclusive association with metastasis (Fig. S3E and S3F). Therefore, we defined these genes as the metastasis-specific neutrophil signature (MNsig) as a potential hallmark of metastasis-induced gene program (Table S4).

Notably, although most prominent in the MN_L_ subset, MNsig was also expressed across other neutrophil subsets in the lung metastatic TME (Fig. S3A and S3G). This broad expression pattern suggests that MNsig represents a conserved gene program induced by the metastatic condition itself, rather than being a marker of one specific subset. Corroborating its link to disease progression, the overall expression of MNsig increased from early dissemination through advanced stages of metastasis (Fig. 3J). The signature's high specificity and its dynamic expression during the metastatic cascade position it as a potential tissue-specific biomarker for lung metastasis.

The genes of the MNsig are enriched in metabolic and structural regulation. Their signature was characterized by genes involved in metabolic processes, particularly glycolysis and pyruvate metabolism, which are often up-regulated to support the energetic demands of the metastatic cancer cells (Fig. S3H) (Welch and Hurst, 2019). Additionally, this signature was also enriched with cystatin genes (*e.g.*, *Cstdc5*, *Stfa2*) linked to protease inhibition, which suggests that MN neutrophils may modulate the extracellular matrix to promote metastasis.

### The MNsig is a conserved feature of neutrophils in the lung metastatic niche

To determine if the MNsig was a general feature of lung metastasis beyond our initial model, we investigated its expression in neutrophils from lung metastases derived from diverse primary tumors (Fig. 3K).

Breast cancer is a major cancer type resulting in lung metastasis.^3^ We first investigated whether neutrophils from lung metastasis of other breast cancer models exhibited this signature. We analyzed public scRNA-seq data from two additional orthotopic breast cancer models, 4T1 and AT3 ^30^. In both models, neutrophils isolated from lung metastases showed elevated expression of the MNsig compared with those from normal lungs (Fig. 3L). Further corroborating this, analysis of bulk RNA-seq performed on neutrophils isolated from the lung metastatic niche of KEP mice also revealed a robust up-regulation of the MNsig genes in a genetically engineered mouse model of spontaneous breast cancer (Fig. S4B).^32^ These data collectively establish that the MNsig is a conserved neutrophil signature within the lung metastatic niche across diverse breast cancer subtypes.

Next, we examined whether other cancer types induced metastasis-specific neutrophils in the lung metastatic niche. First, we analyzed the public scRNA-seq data from an orthotopic model of rhabdomyosarcoma (M3-9-M) that spontaneously metastasizes to the lungs.^31^ Notably, neutrophils within the lung already exhibited MNsig up-regulation at a pre-metastatic stage (day 15 post-tumor inoculation),^31^ suggesting that this program is activated early during metastatic niche formation (Fig. 3M). This finding is consistent with our observations in the early-stage lung metastasis of the MMTV-PyMT model (Fig. 3J).

Similarly, in the B16-F10 experimental metastasis model, our scRNA-seq analysis of neutrophils revealed a robust up-regulation of the MNsig program as early as one week, which progressively increased through the fourth week (Fig. 3N). This temporal dynamic is highly consistent with the trend observed in the spontaneous MMTV-PyMT model, where the MNsig was also up-regulated during the early stages of lung metastasis (Fig. 3J). Because this experimental model isolates the process of tumor cell colonization in the absence of a primary tumor, this observation suggests that the induction of the MNsig is not dependent on systemic signals from an established primary tumor, but is instead possibly induced locally in neutrophils within the lung microenvironment in response to tumor colonization.

Together, the data from sarcoma and melanoma models indicate that the MNsig is not specific to a particular cancer type. Instead, it represents a conserved transcriptional program activated early in the metastatic stage by the lung microenvironment itself during metastatic colonization. This suggests that the MNsig may serve as a potential biomarker for lung metastasis originating from diverse cancer types.

### STEAP4 is a highly metastasis-specific membrane protein in both mouse and human metastatic niches

**Figure 4:**
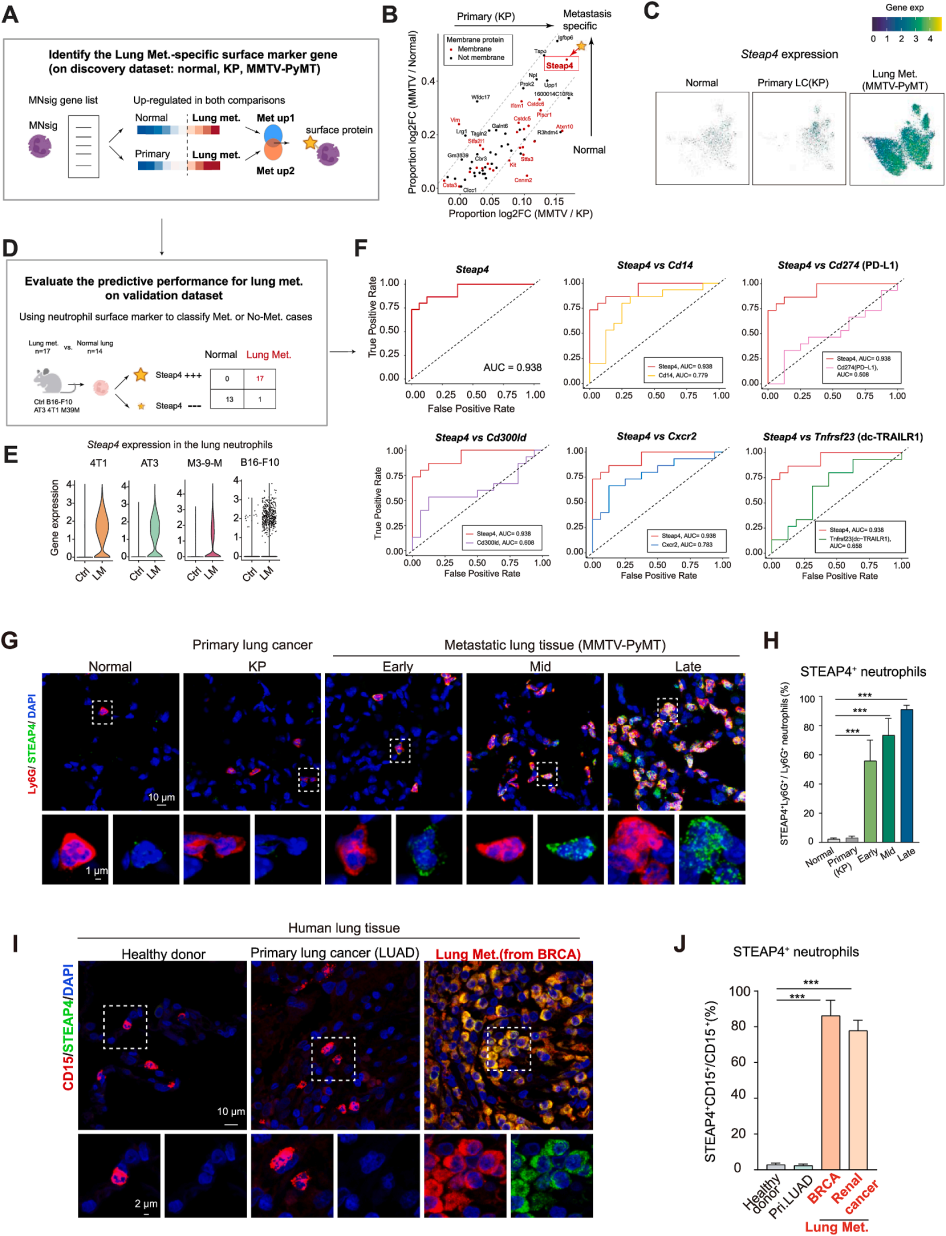
STEAP4, a membrane protein identified as a promising predictive marker for lung metastases. **(A)** Strategy of identification of metastasis-specific surface protein marker genes in the discovery data from the MMTV-PyMT and KP models. **(B)** Scatter plot depicting the metastasis specificity of genes in the MN4 signature compared with normal lung tissue and primary lung tumor in KP. The proportion-weighted log_2_ fold change (log_2_FC) was calculated by combining the value of the mean expression proportion in neutrophils from MMTV-PyMT samples with the log_2_FC of the mean expression proportion across each group of samples. Membrane proteins are highlighted in red. **(C)** Uniform manifold approximation and projection (UMAP) plots showing the *Steap4* expression level in neutrophils from normal lungs, primary lung tumor, and lung metastatic niche. **(D)** Schematic depicting the approach for evaluating *Steap4* as a predictor for lung metastasis. The analysis incorporates validation datasets from B16-F10, AT3, 4T1, and M3-9-M models, compared with normal controls. **(E)** Violin plot showing *Steap4* expression level across different lung metastasis models in the validation datasets in (D). **(F)** The ROC curves measuring the performance of the expression percentage of each membrane protein on neutrophils in predicting lung metastasis using validation datasets in (D). **(G)** Immunofluorescence staining using antibodies against Ly6G and STEAP4 of the lungs from normal, KP, and MMTV-PyMT (early, mid, and late stage) models. Scale bar, 10 μm or 1 μm. **(H)** Percentage of STEAP4^+^ neutrophils in total Ly6G^+^ neutrophils shown in (G). Data were expressed as mean ± standard error of the mean (*n* = 3). ∗∗∗*p* < 0.001 by Student's *t*-test. **(I)** Representative immunofluorescence staining of CD15 and STEAP4 in the lung tissues from healthy donors, lung adenocarcinoma patients, and a breast cancer patient. Scale bar, 10 μm or 2 μm. **(J)** Percentage of STEAP4^+^ neutrophils among total CD15^+^ neutrophils in the sections from healthy donors and patients with lung metastases (mean ± standard deviation, regions = 8, 8, 8, and 4 for healthy donors, patients with lung cancer, breast cancer, and renal cancer). ∗∗∗*p* < 0.001 by Student's *t*-test.

To advance the clinical translation of our findings on metastasis-related biomarkers, we aimed to identify a conserved cell surface protein from the MNsig gene program to enable practical clinical detection. Surface markers are prioritized over intracellular targets as they permit rapid, cost-effective analysis via flow cytometry—a widely available clinical tool. Crucially, an ideal marker must not only distinguish metastatic patients from healthy individuals but, more importantly, differentiate them from those with localized primary lung tumors. Current image-based diagnosis could fail to clearly discriminate between primary lung tumors and metastatic lesions, which disturbs clinical decision-making.^44^^,^^45^ Therefore, we compared the expression percentage of each gene in the MNsig in lung metastases of the MMTV-PyMT model, against those in normal lung tissues or primary lung tumors of the KP model (Fig. 4A). This dual-comparison strategy highlighted *Steap4* as the top candidate cell surface protein, exhibiting exceptional specificity for lung metastasis (Fig. 4B). *Steap4*, a metalloreductase involved in iron and copper homeostasis, may play an important role in the cellular response to inflammatory stress.^46^, ^47^, ^48^ We next examined the expression of *Steap4* in our discovery dataset—the lung neutrophils of the KP and MMTV-PyMT models—and found that *Steap4* was highly specific to the metastatic niche, being broadly expressed across neutrophil subsets within the metastasis-bearing mice but absent in neutrophils originating from the normal lung or primary lung tumors (Fig. 4C). Besides, *Steap4* expression was highly restricted to the neutrophil population among the immune compartment (Fig. S4A).

To determine whether *Steap4* expression is a conserved feature of lung metastasis, we examined its expression in the cross-model validation dataset, including the 4T1, AT3, M3-9-M, and B16-F10 datasets used above (Fig. 3L–N). Consistent with the pattern of MNsig, *Steap4* expression was uniformly up-regulated in neutrophils from the lung metastatic niche across all models (Fig. 4E). Furthermore, bulk RNA-seq data from neutrophils within the lung metastatic niche of KEP mice confirmed the up-regulation of *Steap4* (Fig. S4B).^32^ This remarkable consistency underscores its potential as a robust biomarker for lung metastasis.

Building on its potential as a biomarker, we next sought to quantify the predictive performance of *Steap4* for lung metastasis. We focused this analysis on the pre-clinical mouse models, given the inherent difficulty of studying the nascent pre-metastatic niche in patients (Fig. 4D). To do this, we pooled neutrophil data from the four validation models (Fig. 4D), creating a cohort of metastatic (*n* = 17) and normal (*n* = 14) lung samples. Although tumor-associated neutrophils expressed multiple immunosuppressive cell surface membrane proteins, their utility in distinguishing metastasis remains limited. These cell surface proteins include PD-L1,^49^ CD14,^50^ CD300LD,^51^ dcTRAIL-R1 (*Tnfrsf23*),^12^ CXCR2,^52^^,^^53^ VISTA(*Vsir*),^54^ and CD39 (*Entpd1*).^55^ A direct comparison demonstrated that among these proteins, only *Steap4* consistently showed high expression in lung metastases (Fig. S4C). Strikingly, ROC analysis revealed that *Steap4* exhibited unparalleled performance (AUC = 0.938, 95%CI: 0.86–1.00, *p* < 0.0001), surpassing established markers like *Cd14* (AUC = 0.78, 95%CI: 0.61–0.95, *p* < 0.01), and outperforming the known immunosuppressive genes like *Cd274* (PD-L1) and *Cd3*00ld. Though dcTRAIL-R1, VISTA and CD39 (*Entpd1*) are enriched in tumor-associated neutrophils or macrophages,^12^^,^^54^^,^^55^ they also showed no predictive value for lung metastasis (Fig. 4F; Fig. S4D). This finding was particularly notable as the cohort included samples from the pre-metastatic stage of the M3-9-M model^31^ and B16-F10 experimental metastasis model, highlighting the sensitivity and robustness of *Steap4* as a biomarker.

To further validate STEAP4 on neutrophils at the protein level, we performed immunofluorescence staining on lung tissues from MMTV-PyMT mice. As predicted, STEAP4 was specifically expressed on mouse neutrophils within lung metastases, and staining was absent in normal lung tissues and primary lung tumors (Fig. 4G and H). Further, the proportion of STEAP4^+^ neutrophils emerged at the early-stage metastasis, and was significantly elevated as the lung metastasis progressed (Fig. 4G and H). These results demonstrate that the expression of STEAP4 is strongly associated with metastasis progression, positioning STEAP4^+^ neutrophils as a potential predictive marker for the occurrence of lung metastasis.

To determine whether STEAP4^+^ neutrophils were similarly specific to lung metastases in human patients, we performed immunofluorescence staining for STEAP4^+^ neutrophils in human lung tissue sections. In lungs from both healthy and LUAD patients, STEAP4^+^ neutrophils were rarely detected (Fig. 4I). In contrast, in the lung metastatic lesions from a patient with prior primary breast cancer, the proportion of STEAP4^+^ neutrophils within CD15^+^ neutrophils was significantly elevated, as observed in eight randomly selected regions (Fig. 4I and J). Similarly, lung metastasis in a patient with primary renal cancer also showed a striking increase in STEAP4^+^ neutrophils based on analysis of four randomly selected regions (Fig. 4J; Fig. S5A). Together, these findings demonstrate that STEAP4^+^ neutrophils uniquely emerge within lung metastatic niches in both mouse and human cancers, highlighting a conserved neutrophil response to lung metastases.

### STEAP4^+^ neutrophils as a promising blood-based biomarker for lung metastasis

Neutrophils are highly responsive and sensitive immune cells,^56^ making them uniquely suited to serve as early detectors of disease using immune cells. However, their potential as biomarkers for metastasis remains largely unexplored. Having identified STEAP4^+^ neutrophils within lung metastatic niches, we next investigated whether these neutrophils were confined to local metastatic sites, or could circulate in the blood—a feature that would make them a promising liquid-biopsy biomarker to assist in lung metastasis diagnosis.

**Figure 5:**
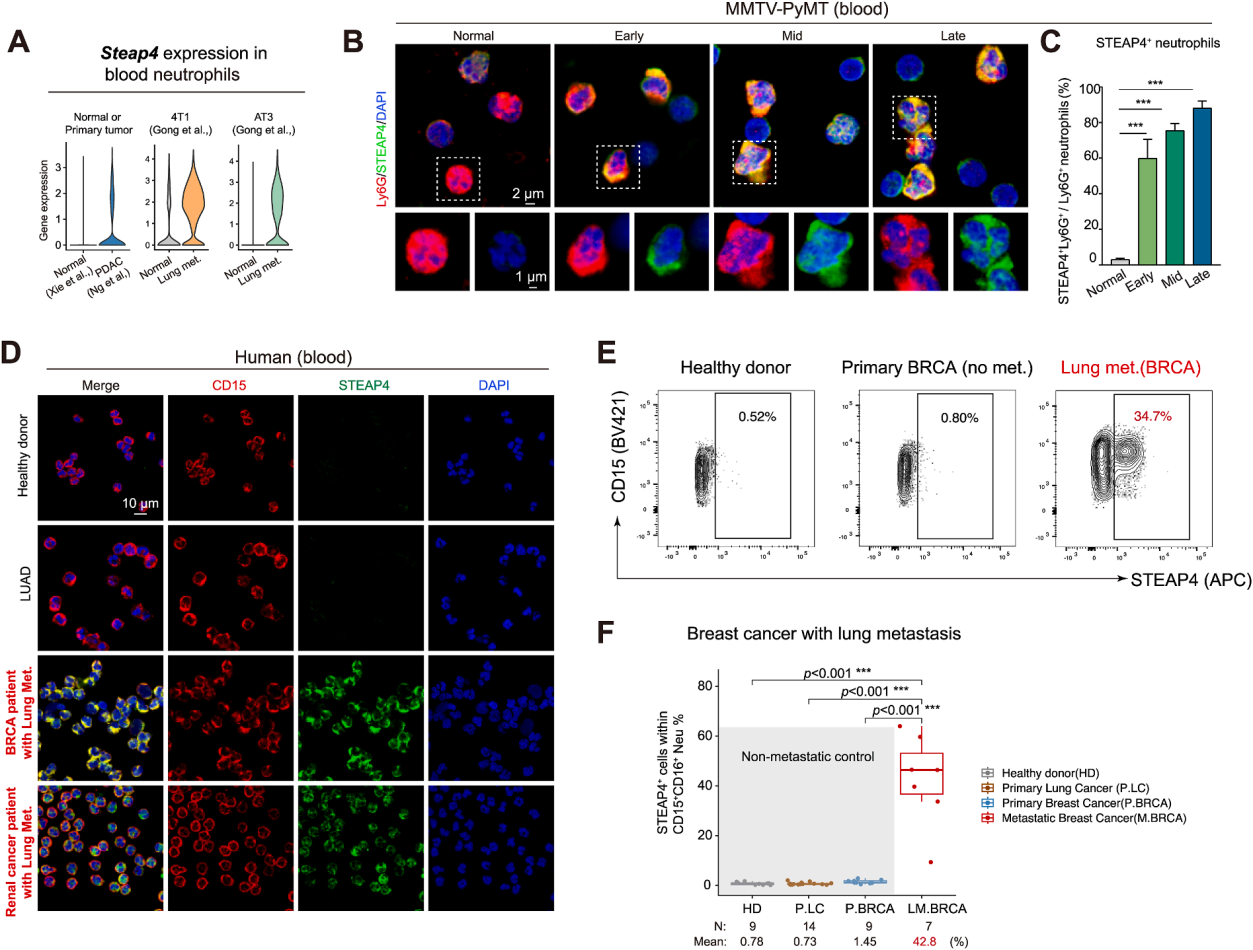
STEAP4^+^ neutrophils are uniquely elevated in the peripheral blood of human cancer patients with lung metastases. **(A)** Violin plot comparing *Steap4* expression in blood neutrophils from normal, primary, and metastatic states across different mouse models. **(B)** Immunofluorescence staining using antibodies against Ly6G and STEAP4 of whole blood cells from normal and MMTV-PyMT (early, mid, late) states. Scale bar, 2 μm or 1 μm. **(C)** Percentage of STEAP4^+^ neutrophils in total Ly6G^+^ neutrophils shown in (B). Data were expressed as mean ± standard error of the mean (*n* = 3). ∗∗∗*p* < 0.001 by Student's *t*-test. **(D)** Peripheral blood neutrophils from both healthy donors and lung metastasis patients were isolated and smeared. Representative immunostaining images of CD15 and STEAP4 in the blood of healthy donors (*n* = 10), lung adenocarcinoma (LUAD) patients (*n* = 10), and patients with lung metastases (*n* = 9). Scale bars, 10 μm. **(E)** Representative flow cytometry scatter plots showing the percentage of STEAP4^+^ cells within CD15^+^CD16^+^ blood neutrophils from healthy donors (*n* = 7), breast cancer (BRCA) patients without metastasis (*n* = 4), and BRCA patients with lung metastases (*n* = 4). Cells were selected based on gating strategies shown in Figure S5C. **(F)** Box plot comparing the percentages of STEAP4^+^ cells within the CD15^+^CD16^+^ neutrophil population among healthy donors (*n* = 9), patients with primary lung cancer (*n* = 14), patients with primary breast cancer (*n* = 9), and breast cancer patients with lung metastases (*n* = 7). Student's *t*-test. Also shown in Table S5.

To assess the presence of STEAP4^+^ neutrophils in the blood, we first analyzed *Steap4* expression in mouse models at the RNA level. Analysis of blood neutrophils via scRNA-seq demonstrated consistently elevated *Steap4* expression in the 4T1, AT3, and KEP mouse models of lung metastasis compared with neutrophils from either non-tumor-bearing mice or a non-metastatic, primary tumor model of orthotopic pancreatic cancer (Fig. 5A; Fig. S5B).^12^^,^^30^^,^^32^ These findings were corroborated by immunofluorescence staining of whole blood cells, which demonstrated that STEAP4^+^ neutrophils were uniquely detectable in blood neutrophils from mice bearing lung metastases. Importantly, these circulating STEAP4^+^ neutrophils emerged at the early-stage metastasis in the MMTV-PyMT model, with their frequency progressively increasing as metastasis progressed (Fig. 5B and C)—a pattern that mirrored the dynamics observed within the lung metastatic niche (Fig. 4G and H). This observation suggests that STEAP4^+^ neutrophils show potential as an early-detection biomarker for lung metastasis.

Next, we examined whether STEAP4^+^ neutrophils could also be specifically detected in the blood of patients with lung metastases. We employed immunofluorescence staining on blood smears as a rapid method to assess the presence of these cells. The staining revealed that both healthy donors (*n* = 10) and primary lung cancer patients (*n* = 10) exhibit minimal STEAP4^+^ neutrophils in the blood. In contrast, lung metastases from multiple cancer types exhibited highly expressed STEAP4 protein on blood neutrophils (Fig. 5D; Table S5), despite their diverse tumor origins. These findings suggest that STEAP4^+^ neutrophils detected in the blood may mirror the metastatic remodeling occurring in lung parenchyma, underscoring that STEAP4^+^ neutrophils may serve as a promising blood-based biomarker capable of discriminating patients with lung metastases from those with primary lung tumors and healthy ones.

We next employed flow cytometry on fresh blood samples to quantitatively investigate whether STEAP4^+^ neutrophils exhibited a distinct abundance in breast cancer patients with lung metastases, compared with non-metastatic breast cancer patients, primary lung cancer patients, and healthy donors (Cohort 2; Table S5). Neutrophils were selected and stained with anti-CD15 and anti-CD16 antibodies (Fig. S5C). In healthy donors (*n* = 9), STEAP4^+^ neutrophils constituted a rare subset of neutrophils (mean, 0.78%) (Fig. 5E and F; Fig. S5D). A similar low proportion was observed in patients with non-metastatic primary tumors, including lung cancer (mean, 0.73%; *n* = 14) and breast cancer (mean, 1.45%, *n* = 9). In contrast, the proportion of circulating STEAP4^+^ neutrophils was significantly elevated in the breast cancer patients with lung metastases, which accounted for a mean of 43% (*n* = 7) of the neutrophil population—a nearly 30-fold increase over patients with primary breast cancer (Fig. 5E and F; Fig. S5D).

## Discussion

In summary, our integrated pre-clinical and clinical analysis provides a comprehensive temporal map depicting the dynamic immune remodeling during lung metastasis. Diverging from the dynamic, lymphocyte-engaged landscape of primary lung tumors, we found that lung metastases—regardless of their primary tumor origins—consistently established a profoundly T-cell-suppressed microenvironment. Concurrently, by systematically comparing the transcriptomic states of neutrophils across primary and metastatic tumors in multiple cancer models, we identified a strictly metastasis-specific neutrophil gene program (MNsig). Crucially, the emergence of this program is highly concurrent with the pathological remodeling of the lung metastatic microenvironment, representing a state unique to metastasis that is distinct from the phenotypes observed in primary tumors or healthy lung tissues. The validation of this signature across diverse cancer models (breast cancer, melanoma, and sarcoma) indicates a fundamental, highly conserved biological adaptation to metastatic colonization. Within this broader context of immune remodeling, we identified STEAP4^+^ neutrophils as a specific manifestation of this conserved adaptation, capable of being detected not only in the local metastatic niche but also in the circulation of breast cancer patients with lung metastasis.

This discovery aligns with the established concept that neutrophils acquire context-specific gene programs to adapt to different pathological conditions.^13^ While both anti-tumoral and pro-tumoral neutrophil subsets have been described in primary tumors,^12^^,^^40^^,^^57^ the gene programs specific to metastasis remain poorly understood. Our findings bridge this critical gap by demonstrating that the metastatic lung environment imprints a distinct, conserved phenotypic state on neutrophils. Because this MNsig is universally up-regulated across diverse pre-clinical models, it potentially highlights an organ-specific, tumor-irrelevant immune response associated with the established metastatic state.

The unique biology of these metastasis-associated neutrophils also offers exciting translational potential. Neutrophils are highly abundant in circulation and highly adaptable, and act as some of the earliest responders to metastatic changes.^42^^,^^58^ Notably, STEAP4^+^ subsets emerge as early as 8 weeks in our MMTV-PyMT model. Furthermore, we observed an up-regulation of the STEAP4-associated signature in neutrophils within the B16-F10 experimental model—which isolates the process of tumor cell colonization in the absence of a primary tumor—and the orthotopic rhabdomyosarcoma model (M3-9-M) at a pre-metastatic stage (day 15 post-tumor inoculation).^31^ This consistent early emergence across distinct models strongly suggests that this specific neutrophil reprogramming initiates during the nascent phases of lung metastasis. Current liquid biopsy approaches predominantly rely on circulating tumor cells, which face critical limitations due to their rarity and heavy reliance on tumor-specific mutation profiles.^9^ Monitoring systemic immune adaptations represents a conceptually distinct and complementary avenue. Rather than proposing it as an immediate standalone diagnostic tool, our work highlights that monitoring systemic neutrophil plasticity—specifically the emergence of STEAP4^+^ neutrophils—represents a potential circulating indicator of the immunosuppressive remodeling occurring in the distant metastatic niche.

We acknowledge that our current study has limitations, particularly the lack of functional validation regarding the specific mechanistic role of STEAP4^+^ neutrophils in driving metastasis or immune suppression. Whether these STEAP4^+^ neutrophils actively orchestrate metastatic progression or are primarily bystander products of the niche remains an open question. Establishing STEAP4 as a definitive therapeutic target or a routine clinical biomarker will strictly require extensive functional studies, *in vivo* depletion/inhibition assays, and validation in larger, multi-center clinical cohorts encompassing diverse primary cancer types (*e.g.*, melanoma, sarcoma, gastrointestinal cancers) to definitively prove its clinical predictive accuracy and generalizability.

While our single-cell trajectory analysis suggests that the emergence of STEAP4^+^ neutrophils represents a process of local microenvironmental adaptation—where recruited early neutrophils undergo terminal differentiation within the lung metastatic niche—their precise developmental origin remains incompletely defined. It is challenging to definitively distinguish whether this STEAP4^+^ reprogramming is entirely driven by local cues within the metastatic lung or if it originates from a specialized bone marrow-derived lineage primed by systemic tumor factors.^32^^,^^59^ This complexity is further underscored by the highly dynamic nature of neutrophils, which can undergo "reverse migration" and re-enter the circulation after tissue education.^60^^,^^61^ Therefore, the STEAP4^+^ neutrophils detected in the peripheral blood could potentially represent a population that was locally educated within the metastatic niche before re-entering the vasculature. Future studies utilizing sophisticated *in vivo* lineage tracing and real-time intravital microscopy will be essential to definitively map their developmental origins and dynamic trafficking.

Furthermore, to comprehensively determine its clinical adaptability as a metastasis indicator, future studies must rigorously evaluate STEAP4 expression across a spectrum of non-cancerous clinical conditions, such as acute infections and chronic inflammatory diseases. Given the inherent sensitivity of neutrophils to generalized stress,^13^^,^^62^ delineating the specificity boundaries of STEAP4 against these inflammatory confounders is critical for its future clinical translation. Although our current study does not position STEAP4^+^ neutrophils as an immediately applicable clinical biomarker, uncovering this conserved, circulating manifestation of the metastatic niche offers a conceptually distinct avenue for biomarker development. Ultimately, by charting the divergent immune trajectories of lung metastasis and identifying this conserved neutrophil program, our work provides a critical biological roadmap for future mechanistic investigations and the development of non-invasive diagnostic strategies.

## Data availability

The gene expression data of this paper can be obtained from the NCBI GEO database with accession number GEO: GSE276180. The raw data can be obtained with GSA: CRA018711 at Genome Sequence Archive in National Genomics Data Center (https://ngdc.cncb.ac.cn/gsa). The data collected from public studies were listed in Table S7. Our analysis code has been uploaded to GitHub (https://github.com/AstreChen/lungmet). We have built a user-friendly interactive website (http://lungmet.cancer-pku.cn/) to help researchers explore and utilize our data.

## Declaration of generative AI and AI-assisted technologies in the manuscript preparation process

During the preparation of this work, C.A. used ChatGPT and Gemini solely to improve the clarity and readability of the English text. No AI tools were used to generate scientific content or data. We reviewed and edited the content as needed and take full responsibility for the content of the published article.

## Funding

This project was supported by funding from the 10.13039/501100012166National Key Research and Development Program of China (No. 2023YFF1204700), the National Natural Science Foundation of China (No. 92459001, 92374205, 82522001, 82470057, 82500086), Shanghai Sailing Program (China) (No. 24YF2722400, Shanghai Oriental Talent Program (China) (No. QNWS2024087), and the Noncommunicable Chronic Diseases-National Science and Technology Major Project (No. 2025ZD0552500).

## Conflict of interests

Z.Z. is a founder of *Analytical Biosciences*. The other authors declare no competing interests.

## Appendix A Supplementary data

Multimedia component 2

Table S1 Clinical characteristics of human subjects analyzed by immunofluorescence staining.

Table S2 Murine samples for scRNA-seq in this study.

Table S3 T cell activation signatures (related to Figure S1).

Table S4 Metastasis-specific signature in neutrophils.

Table S5 Clinical characteristics of human subjects providing fresh blood samples for flow cytometry analysis.

Table S6 Marker genes for neutrophil subsets in scRNA-seq analysis.

Table S7 The published data used in this paper.
