# Supplementary material for: Dynamic single-cell landscape reveals a T-cell-suppressed microenvironment and a conserved neutrophil program in the lung metastatic niche
Source: Genes Dis. 2026 Apr 27;13(6):102214. doi: 10.1016/j.gendis.2026.102214 (PMC13487611; doi:10.1016/j.gendis.2026.102214)

Figure S1. Immune status of lung metastatic TME in humans and mice.

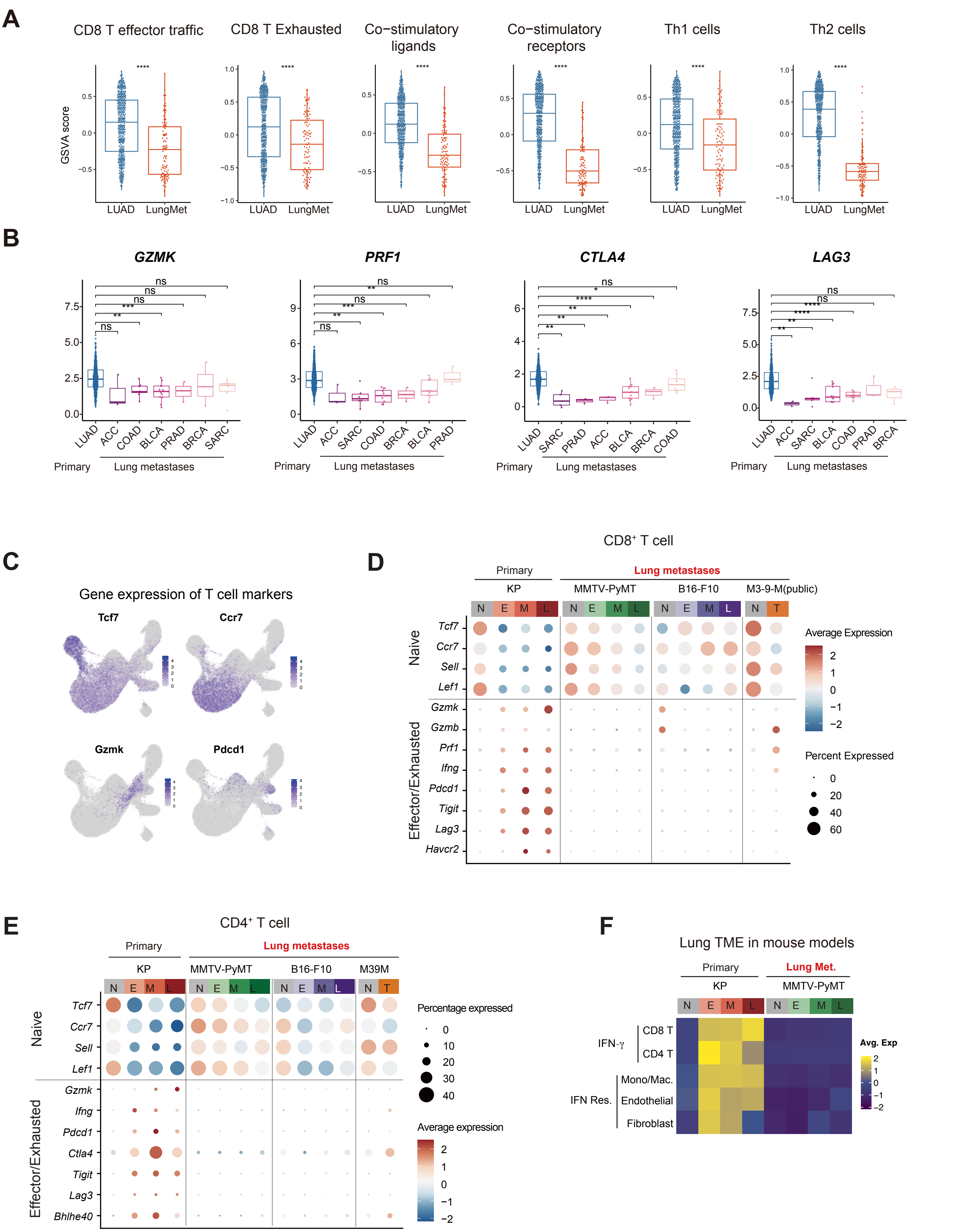

Figure S2. Dynamic changes of cell types in primary lung tumors and lung metastases.

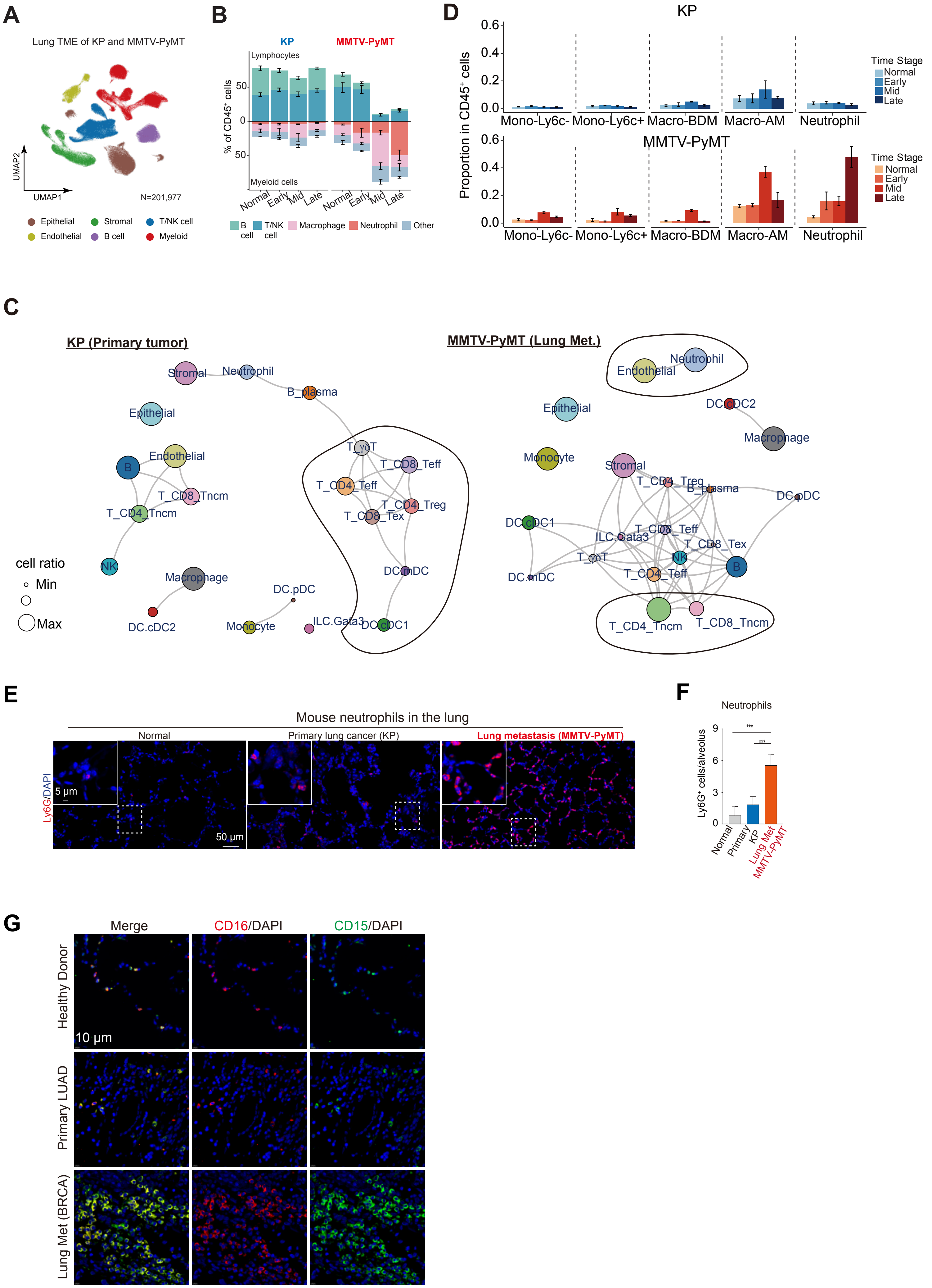

Figure S3. Characterization of neutrophils and MNSig via scRNA-seq analysis.

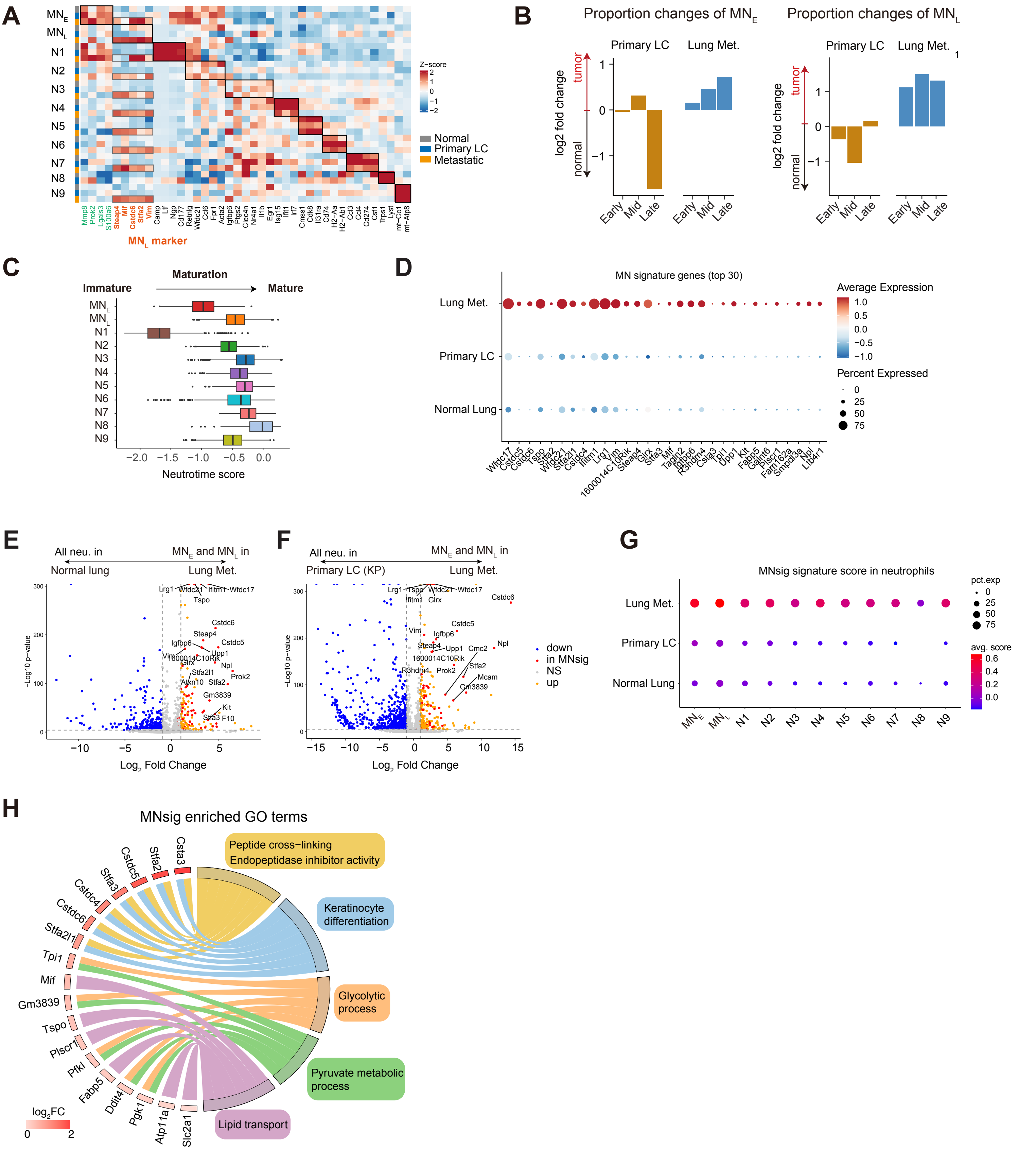

Figure S4. Validation of the specificity of STEAP4+ neutrophils in the lung metastases of mouse models.

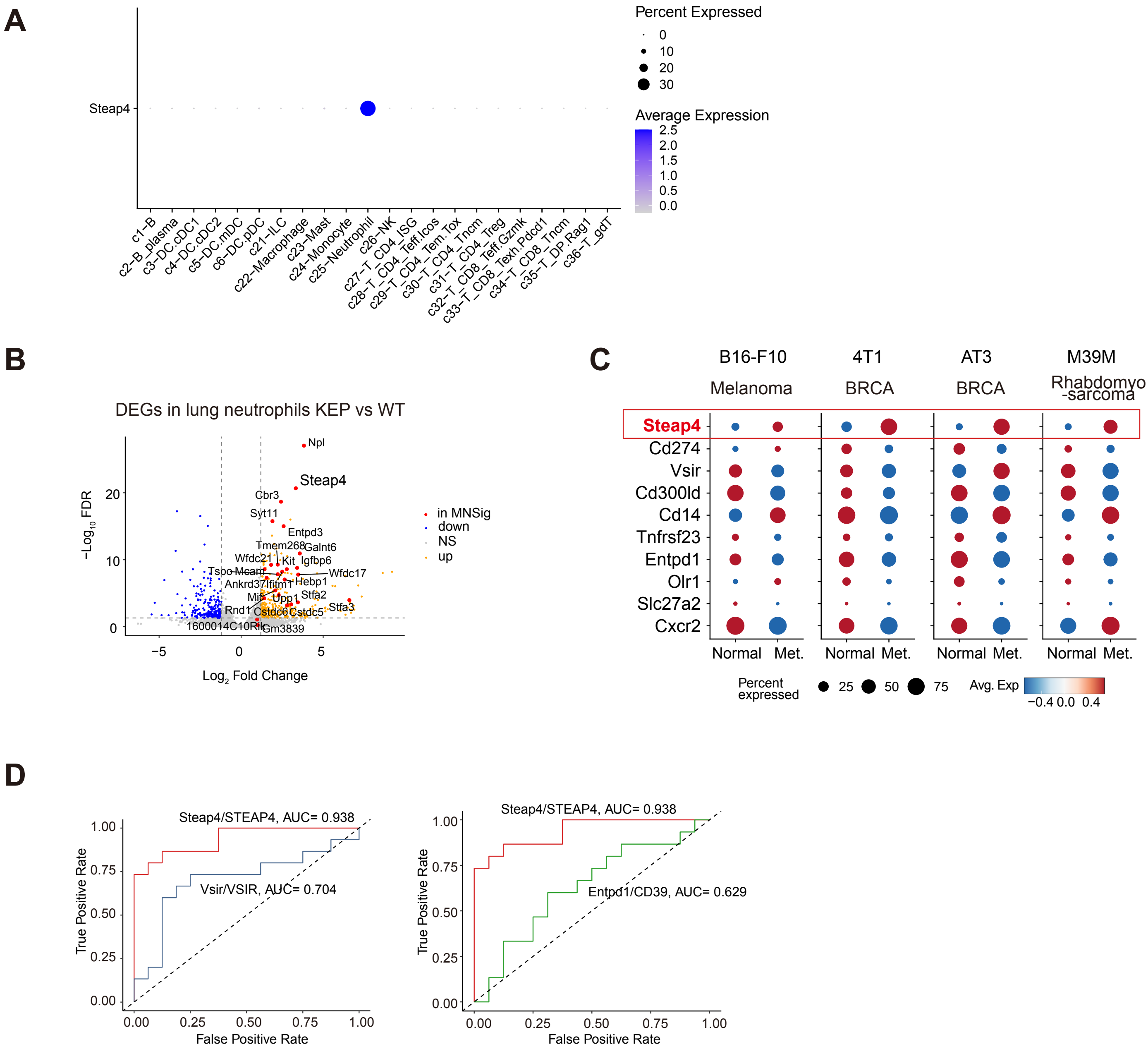

Figure S5. Confirmation of STEAP4⁺ neutrophils in peripheral blood.

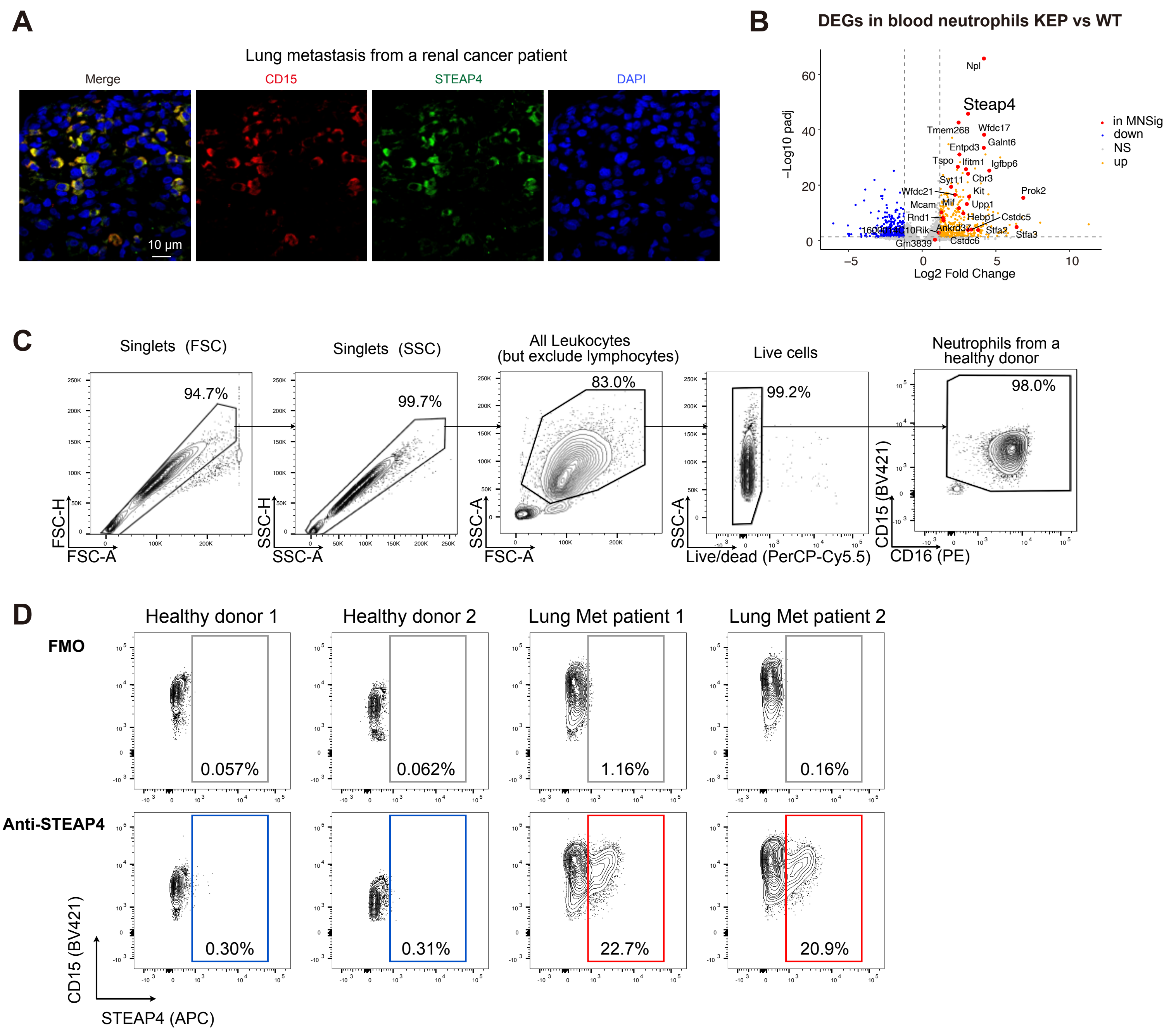

Supplement: Supplementary file 1 [file mmc2.pdf]
