# Supplementary material for: Dynamic single-cell landscape reveals a T-cell-suppressed microenvironment and a conserved neutrophil program in the lung metastatic niche
Source: Genes Dis. 2026 Apr 27;13(6):102214. doi: 10.1016/j.gendis.2026.102214 (PMC13487611; doi:10.1016/j.gendis.2026.102214)
Supplement: Supplementary file 2 [file mmc1.docx]

**Figure S1** Immune phenotypes of lung metastases in human patients and mouse models. **(A)** Box plots showing differences in the activity of six T cell signatures between lung primary tumors (*n* = 931) and metastatic tumors (*n* = 100). ^*^*P* < 0.05, ^**^*P* < 0.01, and ^***^*P* < 0.001 by two-sided *t*-test. **(B)** Box plots showing the expression level of effector (*GZMK*, *PRF1*) and checkpoint molecules (*CTLA4*, *LAG3*) in patients with primary lung cancer and patients with lung metastasis. ^*^*P* < 0.05, ^**^*P* < 0.01, ^***^*P* < 0.001, ^****^*P* < 0.0001, and ns (not significant) by Student's *t*-test. **(C)** Uniform manifold approximation and projection (UMAP) plots illustrating the expression levels of canonical marker genes for naïve T cells (*Tcf7* and *Ccr7*) and activated T cells (*Gzmk* and *Pdcd1*). **(D, E)** Bubble plot showing expression level of canonical marker genes in CD8^+^ and CD4^+^ T cells from lungs in mouse models of primary and metastatic lung tumors. Label of samples: N, normal; E, early; M, mid; L, late; T, tumor. **(F)** Heatmap showing the scaled gene expression of *Ifng* in CD8^+^ and CD4^+^ T cells, and the signature score of response to interferons of GO terms in lung tissue samples from different time points of mouse models. Label of samples: N, normal; E, early; M, mid; L, late.

**Figure S2** Dynamic changes of cell types in primary lung tumors and lung metastases. **(A)** Uniform manifold approximation and projection (UMAP) plot of the major cell types from the single-cell atlas of lung microenvironment from MMTV-PyMT and KP models. NK cells, natural killer cells. **(B)** Frequencies of major immune cell populations (as a percentage of CD45^+^ cells) in KP and MMTV-PyMT mouse lungs. **(C)** Cellular correlation networks in KP primary tumors (left) and MMTV-PyMT lung metastases (right). Nodes represent distinct cell types, with sizes reflecting their relative proportions within the CD45^+^ or CD45^–^ fractions. Edges connect cell types with a pairwise correlation >0.5 based on the sample × cell type frequency matrix. **(D)** Frequencies of major immune cell populations (as a percentage of CD45^+^ cells) in KP and MMTV-PyMT mouse lungs. **(E, F)** Neutrophils were stained with antibodies against Ly6G in the lungs from normal, KP, and MMTV-PyMT mice (E), and their number in the alveoli was quantified (F). Data were expressed as mean ± standard error of the mean (*n* = 3). ^***^*P* < 0.001 by Student's *t*-test. Scale bar, 50 μm or 5 μm. **(G)** Representative immunofluorescence images of neutrophils in human lung tissues from a healthy donor, primary lung adenocarcinoma (LUAD), and lung metastasis from breast cancer (BRCA). Sections were co-stained for CD16 (red) and CD15 (green). Nuclei were counterstained with DAPI (blue). Scale bar, 10 μm.

**Figure S3** Characterization of neutrophils via scRNA-seq analysis. **(****A)** Heatmap showing top marker genes of each neutrophil subset split by conditions shown in Figure 3A. See Table S6 for all top 50 marker genes. **(****B)** Bar plots showing changes in cell proportion of MN_E_ and MN_L_ subsets in primary lung tumors versus metastatic lung tumors via log fold change calculated based on the mean proportion across samples in each group. **(C)** Neutrotime signature score of each subset of neutrophil in the normal, KP, and MMTV-PyMT lungs. The large neutrotime scores represent a mature state. **(D)** Bubble plot depicting expression level and percentage of the MN signature genes in neutrophils from normal lungs, primary lung tumors (KP), and lung metastases (MMTV-PyMT). **(E)** Volcano plot showing the differentially expressed genes between metastasis-specific neutrophils (MN_E_ + MN_L_) in the MMTV-PyMT and neutrophils from normal lungs. **(F)** Volcano plot showing the differentially expressed genes between metastasis-specific neutrophils (MN_E_ + MN_L_) in the MMTV-PyMT and neutrophils in the primary lung tumor (KP model). **(G)** Bubble plot depicting the level and percentage of MNsig score within different lung metastasis mouse models. Scores were calculated using AddModuleScore. **(H)** Chord diagram showing MNsig genes and the enriched pathways in GO terms. The log2 fold change represents the expression change in MN_L_ versus other neutrophil subsets.

**Figure S4** Validation of the specificity of STEAP4^+^ neutrophils in the lung metastases of mouse models. **(A)** Bubble plot depicting the level and percentage of Steap4 within all immune cell types in our single-cell atlas. **(B)** Volcano plot displaying differential gene expression in lung neutrophils from KEP versus wild-type mice. The data were re-analyzed using the bulk RNA-seq data ^32^. **(C)** Bubble plot depicting the level and percentage of the cell surface protein genes within different lung metastasis mouse models. **(D)** The ROC curves showing the performance of the expression percentage of VISTA, CD39, and STEAP4.

**Figure S5** Confirmation of STEAP4^+^ neutrophils in peripheral blood. **(A)** Representative immunostaining of lungs from a renal cancer patient with lung metastases using antibodies against CD15 and STEAP4. Scale bar, 10 μm. **(B)** Volcano plot displaying differential gene expression in blood neutrophils from KEP versus wild-type mice. The data were re-analyzed using the bulk RNA-seq data from ^32^. **(C)** Gating strategies for selecting neutrophils from fresh blood are shown in Figure 5E. **(D)** Representative contour plots showing the frequencies of STEAP4^+^ neutrophils in the healthy donors and patients with metastasis. Cells were selected based on gating strategies shown in Figure S5C and then gated using the fluorescence minus one (FMO) control.

**Table S1** Clinical characteristics of human subjects analyzed by immunofluorescence staining.

**Table S2** Murine samples for scRNA-seq in this study.

**Table S3** T cell activation signatures (related to Figure S1).

**Table S4** Metastasis-specific signature in neutrophils.

**Table S5** Clinical characteristics of human subjects providing fresh blood samples for flow cytometry analysis.

**Table S6** Marker genes for neutrophil subsets in scRNA-seq analysis.

**Table S7** The published data used in this paper.
